# Immunosuppressive Polymeric Nanoparticles Targeting Dendritic Cells Alleviate Lupus Disease in *Fcgr2b*^-/-^ Mice by Mediating Antigen-Specific Immune Tolerance

**DOI:** 10.3390/ijms24098313

**Published:** 2023-05-05

**Authors:** Phuriwat Khiewkamrop, Chamraj Kaewraemruaen, Chonnavee Manipuntee, Chalathan Saengruengrit, Numpon Insin, Asada Leelahavanichkul, Warerat Kaewduangduen, Opor Sonpoung, Kasirapat Ariya-anandech, Nattiya Hirankarn, Patcharee Ritprajak

**Affiliations:** 1 Research Unit in Integrative Immuno-Microbial Biochemistry and Bioresponsive Nanomaterials, Faculty of Dentistry, Chulalongkorn University, Bangkok 10330, Thailand; 2 Center of Excellence in Immunology and Immune-Mediated Diseases, Faculty of Medicine, Chulalongkorn University, Bangkok 10330, Thailand; 3 Graduate Program in Medical Microbiology, Graduate School, Chulalongkorn University, Bangkok 10330, Thailand; 4 Department of Science and Bioinnovation, Faculty of Liberal Arts and Science, Kasetsart University, Kamphaeng Saen Campus, Nakhon Pathom 73104, Thailand; 5 Department of Chemistry, Faculty of Science, Chulalongkorn University, Bangkok 10330, Thailand; 6 Bureau of Quality and Safety of Food, Department of Medical Sciences, Ministry of Public Health, Nonthaburi 11000, Thailand; 7 Translational Research in Inflammation and Immunology Research Unit (TRIRU), Department of Microbiology, Faculty of Medicine, Chulalongkorn University, Bangkok 10330, Thailand; 8 Oral Biology Research Center, Faculty of Dentistry, Chulalongkorn University, Bangkok 10330, Thailand; 9 Immunology Unit, Department of Microbiology, Faculty of Medicine, Chulalongkorn University, Bangkok 10330, Thailand; 10 Department of Microbiology, Faculty of Dentistry, Chulalongkorn University, Bangkok 10330, Thailand

**Keywords:** PDMAEMA-PLGA nanoparticles, dexamethasone, tolerogenic dendritic cells, immune tolerance, lupus disease

## Abstract

Dendritic cells (DCs) are the most potent antigen-presenting cells that have multifaceted functions in the control of immune activation and tolerance. Hyperresponsiveness and altered tolerogenicity of DCs contribute to the development and pathogenesis of system lupus erythematosus (SLE); therefore, DC-targeted therapies aimed at inducing specific immune tolerance have become of great importance for the treatment of SLE. This study developed a new nanoparticle (NP) containing a biodegradable PDMAEMA-PLGA copolymer for target-oriented delivery to DCs in situ. PDMAEMA-PLGA NPs provided sustained drug release and exhibited immunosuppressive activity in FLT3L and GM-CSF-derived bone marrow in conventional DCs (BM-cDCs). PDMAEMA-PLGA NPs improved dexamethasone capability to convert wild-type and *Fcgr2b*^-/-^ BM-cDCs from an immunogenic to tolerogenic state, and BM-cDCs treated with dexamethasone-incorporated PDMAEMA-PLGA NPs (Dex-NPs) efficiently mediated regulatory T cell (Treg) expansion in vitro. Dex-NP therapy potentially alleviated lupus disease in *Fcgr2b*^-/-^ mice by mediating Foxp3^+^ Treg expansion in an antigen-specific manner. Our findings substantiate the superior efficacy of DC-targeted therapy using the PDMAEMA-PLGA NP delivery system and provide further support for clinical development as a potential therapy for SLE. Furthermore, PDMAEMA-PLGA NP may be a versatile platform for DC-targeted therapy to induce antigen-specific immune tolerance to unwanted immune responses that occur in autoimmune disease, allergy, and transplant rejection.

## 1. Introduction

Dendritic cells (DCs) are a diverse group of specialized antigen-presenting cells that possess the ability to integrate information about their environment and communicate it to other leukocytes, leading to activation of immune responses and mediation of immune homeostasis and immune tolerance. Dendritic cells have the bidirectional ability to initiate and dictate immunity towards immune activation and immune tolerance. Immunogenic DCs triggered by inflammatory stimuli play an important role in inducing the effector T cell response and immune activation. On the contrary, anti-inflammatory signals that induce tolerogenic DCs or signals that interfere with immunogenic DC function play a key role in facilitating regulatory T cell (Treg) development and immune tolerance [1,2]. Tolerogenic DCs can be divided into natural tolerogenic DCs, which are natural arising immature DCs, and induced tolerogenic DCs, which are instructed by the immunosuppressive microenvironment or manipulated by using pharmacological and biological agents [1,2]. Both DCs participate in the induction of immune tolerance through various mechanisms such as low levels of co-stimulatory signals, low levels of MHC expression, high levels of inhibitory T cell molecules, and high levels of immunosuppressive mediators [2]. 

Systemic lupus erythematosus (SLE) is a chronic autoimmune inflammatory disease that affects several organs, such as the kidneys, joints, and central nervous system, which is commonly found in women between puberty to menopause [3]. SLE results from loss of immune tolerance to self-antigens leading to aberrant immune responses, high autoantibody production, and immune complex deposition [3]. Non-targeted immunosuppressive treatment has been widely used to attenuate the harmful effects of autoimmunity, allergic immune responses, and responses against transplanted organs. However, this non-targeted immunosuppressive treatment represses the whole immune system, which consequently causes an increased risk of infection in patients. In addition, some immunosuppressants, such as corticosteroids, have many long-term risks and serious side effects. As such, induction of specific immune tolerance can prevent serious complications from long-term use of immunosuppressive drugs. 

An alternative strategy to control a harmful immune response to self-antigens in patients with SLE is the induction of an antigen-specific immune tolerance. As DCs are key regulators of immune tolerance, DC-targeted therapies have garnered a great deal of interest for the treatment of SLE and other autoimmune diseases. A nanoparticle-based drug delivery system offers an alternative solution for the target-oriented delivery of precise medicines. Exploiting nanoparticles (NPs) to target multiple subsets of DCs in situ and harness them into tolerogenic phenotypes represents a promising strategy to mediate antigen-specific immune tolerance in patients with autoimmune disorders [4]. In addition, the use of nanoparticles with immunomodulatory properties can potentially reinforce DC tolerogenicity [5]. To date, few studies have focused on nanoparticle-mediated immune tolerance for the therapy of autoimmune disease therapy, which makes this nanotechnology still in its infancy.

Poly(2-(dimethylamino)ethyl methacrylate) (PDMAEMA) is a hydrophilic cationic vinyl-based polymer that has been widely used in gene delivery because it can form a polyplex with DNA by electrostatic interaction. Polyplexes of PDMAEMA and DNA can destabilize endosomes, resulting in endosomal escape, and DNA is dissociated from PDMAEMA in the cytoplasm, contributing to transfection efficiency [6]. From an in vivo study in a mouse model, PDMAEMA/DNA polyplexes can be uptaken by lung tissues [7]. PDMAEMA also has pH-sensitive properties that are critical for the drug delivery system [8]. The use of PDMAEMA NPs in medical applications is limited due to their molecular architecture and low degradability in vivo; however, modified PDMAEMA and the combination of PDMAEMA with other polymers improved the properties for drug delivery applications [5,7,8]. We previously fabricated PDMAEMA-coated iron oxide nanocubes and investigated these nanocomposite properties in macrophages and DCs in vitro and found that PDMAEMA-coated iron oxide nanocubes exhibited low cellular toxicity and could suppress the inflammatory response in both cells [9,10]. 

Poly(D,L-lactide-coglycolide) (PLGA) is an aliphatic degradable and biocompatible thermoplastic polyester which is one of the best characterized and has been approved by the US Food and Drug Administration (FDA) for the delivery of drugs and biomolecules. Previously, we developed PLGA-based nanoparticles (NPs) for antigen delivery to DCs and demonstrated that PLGA NPs produced low cell toxicity and were biocompatible to DCs; however, PLGA NPs exhibited immunogenic activity that may not be suitable for therapeutic application in autoimmune diseases [11,12]. 

In this context, this study fabricated a novel nanoparticle by modifying PLGA with PDMAEMA to alter the immunological properties of PLGA, and we tested the immunosuppressive activity of PDMAEMA-PLGA NPs in vitro and in vivo by using *Fcgr2b*^-/-^ mice. Polymorphism studies in human *FCGR2B* demonstrated that this receptor is strongly associated with susceptibility to SLE in Caucasians and Southeast Asians [13,14]. *Fcgr2b*-deficient mice correspondingly developed the autoimmune lupus-like disease resembling human SLE. Compared to other mouse models of lupus diseases that have an intact expression of *Fcgr2b* (e.g., NZB/NZW), *Fcgr2b*^-/-^ mice display strong autoimmunity with lupus phenotypes [12]. In addition, *Fcgr2b*^-/-^ mice have highly upregulated autoantibodies, since the *Fcgr2b* allele is associated with increased IgG production [12,15]. Therefore, the use of *Fcgr2b*^-/-^ mice provided insight into a clinical design for the treatment of SLE. Our in vitro findings and the preclinical study confirm the utility of the PDMAEMA-PLGA NP platform as a promising alternative to DC-targeted therapy in the treatment of SLE and other autoimmune diseases. 

## 2. Results

### 2.1. Characterization of Nanoparticles

**Figure 1:**
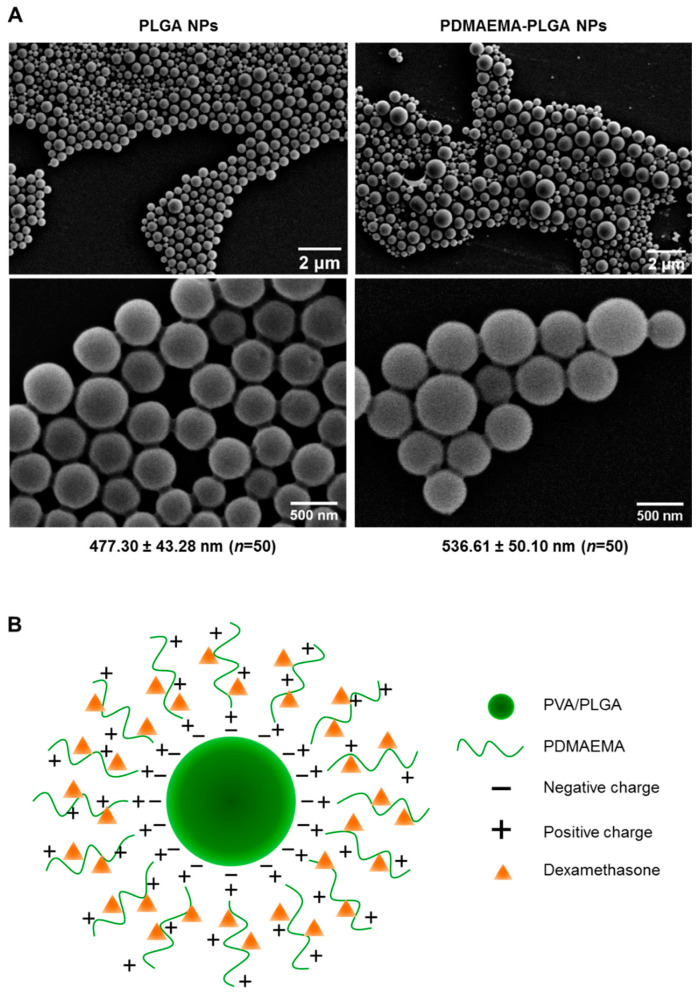
Characterizations of the synthesized nanoparticles. (**A**) Two magnifications of scanning electron microscopy (SEM) images of PLGA NPs and PDMAEMA-PLGA NPs. The numbers indicated the average size of the NPs (mean ± SD) from fifty particles. (**B**) Illustration of dexamethasone-incorporated PDMAEMA-PLGA NPs.

PLGA NPs and PDMAEMA-PLGA NPs were prepared using a single emulsion/solvent evaporation method that was modified from the preparation of PLGA NPs in our previous work [11]. Because the particle size of 500 nm is in a favorable range for active internalization by DCs [11,16], we selected the particle size of 500 nm using stepwise centrifugation of various centrifugal forces [11]. From SEM analysis, PLGA NPs and PDMAEMA-PLGA NPs exhibited spherical shapes with the sizes of 477.30 ± 43.28 nm and 536.61 ± 50.10 nm, respectively (Figure 1A). The hydrodynamic size and the polydispersity index (PDI) of the PLGA NPs (509.14 ± 11.39, PDI 0.185 ± 0.007) and the PDMAEMA-PLGA NPs (547.52 ± 30.04, PDI 0.214 ± 0.019) corresponded to the sizes measured by SEM (Table 1 and Figure 1A). SEM and DLS measurements indicated an increase in size without a significant change in the size distribution after the PLGA NPs were coated with another layer of PDMAEMA. The observation of spherical NPs from SEM after lyophilization indicated the stability of the NPs of both compositions. Incorporation of dexamethasone into the PDMAEMA-PLGA NPs did not affect the hydrodynamic size (541.56 ± 51.85) and PDI (0.198 ± 0.030) of the NPs (Table 1).

The coating of PDMAEMA on PLGA has been well observed as a change in the zeta potential of NPs (Table 1). The zeta potential of the PLGA NPs was −24.5 ± 0.4 mV and these negative charges were probably due to the presence of free carboxylic end groups in the PLGA polymer, as previously proposed that NPs were formed when a PVA-stabilized emulsion with PLGA was exposed to an aqueous phase on the outsides [17]. After the addition of positively charged PDMAEMA in the water phase, the zeta potential of PDMAEMA-PLGA increased to +33.2 ± 0.5 mV. These observations suggested that the cationic polymer PDMAEMA was bound to PLGA by electrostatic interactions and the surface of the PDMAEMA-PLGA NPs was mostly covered by PDMAEMA (Figure 1B). Incorporation of dexamethasone into PDMAEMA-PLGA NPs slightly decreased the surface charges of the NPs to +32.3 ± 0.4 mV. Since dexamethasone in the form of a solution is composed of negatively charged ions [18], the reduced charges of PDMAEMA-PLGA NPs incorporated with dexamethasone may be due to neutralization of the charge by the low amount of dexamethasone that interacted with the positive charges of PDMAEMA (Figure 1B and in the Section 4).

In the TGA and DTA traces of PDMAEMA-PLGA NPs (Figure S1A,B), the materials showed combined characteristics of PDMAEMA, PLGA, and PVA with no significant changes in thermal stability of individual polymers. The materials did not experience significant weight loss up to 200 °C, indicating that the composite NPs exhibited thermal stability similar to that of PDMAEMA. As observed, pure polymers of PLGA and PVA showed a significant weight change from 222 °C and 236 °C, respectively, while PDMAEMA started decomposition at 200 °C. The appearance of degradation of PLGA-PDMAEMA can only be attributed to the degradation of PDMAEMA [19], confirming the existence of PDMAEMA in the composites. Moreover, the DTA trace showed a substantial character of PVA, indicating that PVA plays an important role in the formation of PDMAEMA-PLGA NPs.

### 2.2. PDMAEMA-PLGA NPs Exhibited High Stability and Sustained Drug Release

**Figure 2:**
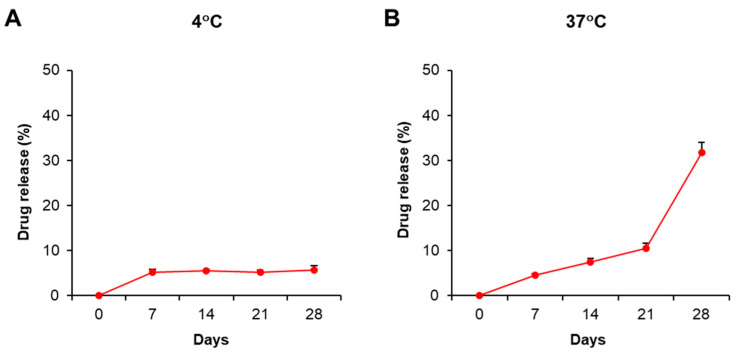
Stability and drug release profiles of dexamethasone-incorporated NPs. The suspension of PDMAEMA-PLGA NPs and dexamethasone-incorporated PDMAEMA-PLGA NPs were incubated at (**A**) 4 °C and (**B**) 37 °C in the dark for 28 days. The supernatants were collected on days 0, 7, 14, and 28 for free dexamethasone measurement with a UV-VIS spectrophotometer. The percentages of drug release were calculated as described in Section 4. The experiments were carried out in triplicate. Free dexamethasone in the supernatants was determined at absorbances of 242 nm and 600 nm. The amounts of free dexamethasone were calculated using the following formula: [(O.D. 242 nm − O.D. 600 nm) of Dex-NPs − (O.D. 242 nm − O.D. 600 nm) of blank NPs] × Extinction coefficient (13.770 × 10^3^/M^−1^/cm^−1^) [20]. The percentages of drug release were calculated by normalizing the amounts of free dexamethasone each day to day 0.

This current study aimed to study the immunosuppressive activity of PDMAEMA-PLGA NPs; therefore, we selected dexamethasone as a model and positive control because dexamethasone exhibits strong immunosuppression. The stability and drug release profiles of PDMAEMA-PLGA NPs were evaluated by the incorporation of NPs with dexamethasone (Dex-NPs). Since pure dexamethasone shows the absorption spectra at λ = 242 nm (Figure S1A) [20], we used UV–VIS spectroscopy to determine the optical property of the free dexamethasone that was released from the samples. The suspension of blank PDMAEMA-PLGA NPs and Dex-NPs were incubated at 4 °C and 37 °C for 28 days and the drug releases were observed weekly (Figure S2 and Figure 2). Dex-NPs incubated at 4 °C showed high stability as free dexamethasone was released from NPs approximately 5–6% from 7 to 28 days (Figure 2A and Figure S2B–F). These results indicated favorable stability of Dex-NPs at low temperature, which is suitable for long storage.

The release profile of free dexamethasone at 37 °C demonstrated that the drug was slowly released and remained controlled for 21 days (approximately 4–10%) and was released faster from 21 to 28 days (adjusted from 10% to 30%) (Figure 2B and Figure S2B–F). The results indicated the release of free dexamethasone was sustained and controlled by PDMAEMA-PLGA NPs.

### 2.3. PDMAEMA-PLGA NPs Exhibited Immunosuppressive Activities and Improved the Tolerogenic Effects of Dexamethasone in Wild-Type BM-cDCs

Conventional DCs are the major DC population of which their development requires the signal from the FMS-like tyrosine kinase 3 ligand (FLT3L) [21]. The additional signal from GM-CSF is also critical for the full differentiation of cDC1 and cDC2 [22,23]. To study the effect of our NPs on cDCs, in vitro cDCs were generated from mouse bone marrow using FLT3L and GM-CSF.

**Figure 3:**
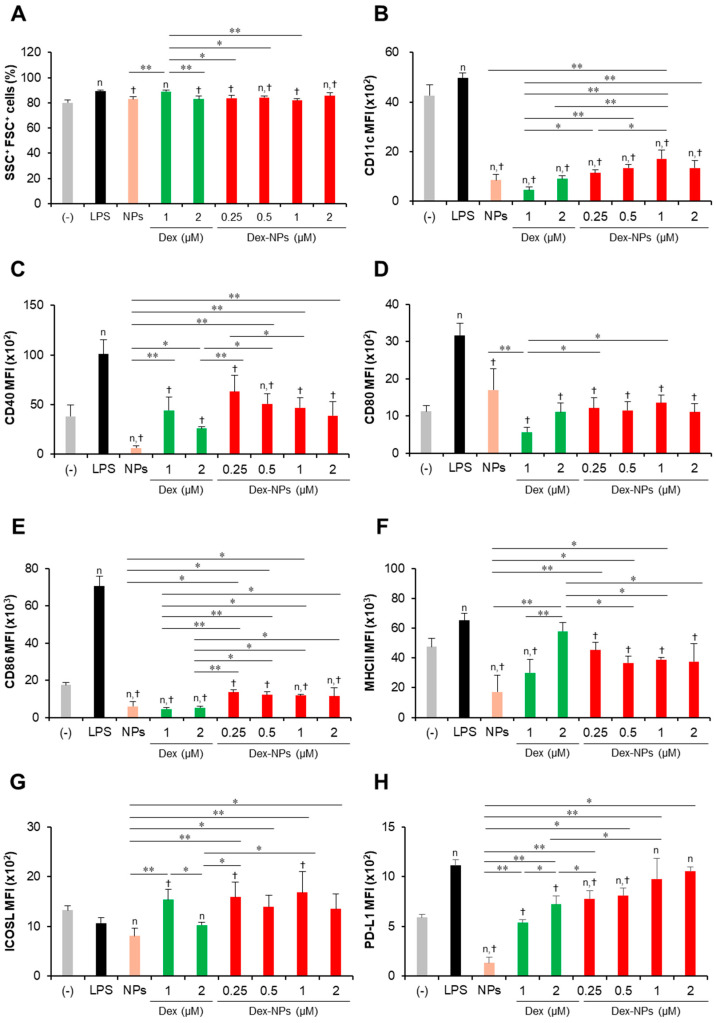
Immunomodulatory effects of PDMAEMA-PLGA NPs and dexamethasone-incorporated PDMAEMA-PLGA NPs on the maturation of wild-type BM-cDCs. Wild-type BM-cDCs (1 × 10^6^ cells/well) were pre-incubated with blank PDMAEMA-PLGA NPs (55 μg, an equal amount to Dex-NPs containing 2 μM dexamethasone), dexamethasone (1 and 2 μM), and dexamethasone-incorporated PDMAEMA-PLGA NPs containing 1 and 2 μM dexamethasone for 48 h. Subsequently, the DCs were stimulated with 0.1 μg/mL of LPS for 24 h. (**A**) The percentages of SSC^+^FSC^+^ cells and the expression of (**B**) CD11c, (**C**) CD40, (**D**) CD80, (**E**) CD86, (**F**) MHC class II, (**G**) ICOSL, and (**H**) PD-L1 were assessed by flow cytometry. *n* = 5; ^n^ *p* ≤ 0.05 compared with the negative control, ^†^ *p* ≤ 0.05 compared with LPS-stimulated BM-cDCs, * *p* ≤ 0.05, ** *p* ≤ 0.001; (−), negative control (untreated and unstimulated BM-cDCs); LPS, LPS-stimulated BM-cDCs; Dex, dexamethasone; Dex-NPs, dexamethasone-incorporated PDMAEMA-PLGA NPs.

To examine the in vitro tolerogenic induction ability of PDMAEMA-PLGA NPs and Dex-NPs in comparison with free dexamethasone, wild-type (WT) BM-cDCs were pretreated with blank PDMAEMA-PLGA NPs, 1 and 2 μM dexamethasone, and Dex-NPs containing 0.25, 0.5, 1, and 2 μM dexamethasone for 48 h. Cells were stimulated with LPS for 24 h and DC activation was determined by flow cytometric analysis (Figure 3). To determine the effects of PDMAEMA-PLGA NPs, dexamethasone, and Dex-NPs on cell size and cellular complexity, the forward scatter (FSC) and side scatter (SSC) plots were analyzed. BM-cDCs treated with blank NPs, dexamethasone, and Dex-NPs showed a proportion of SSC^+^FSC^+^ cells greater than 80% and there was no apparent difference in the number of this population between all samples (Figure 3A), which indicated that blank PDMAEMA-PLGA NPs, dexamethasone, and Dex-NPs did not cause cellular toxicity due to no alteration of cell size and cellular complexity. These results were consistent with the cellular toxicity test, in which the PDMAEMA-PLGA NPs at concentrations ranging from 12.5–100 µg/mL showed no cellular toxicity at 24 h and 48 h after incubation with NPs (Figure S3A,B).

Subsequently, the expression levels of the DC marker, CD11c (Figure 3B), the DC activation markers, CD40, CD80, CD86, and MHC class II (Figure 3C–F) were determined using histogram analysis (Figures S4–S6). Furthermore, the proportions of CD11c^+^, CD40^+^, CD80^+^, CD86^+^, and MHC class II^+^ cells were also analyzed using dot plot analysis (Figures S7–S10). Compared to unstimulated BM-cDCs, LPS-stimulated BM-cDCs matured as the expression of CD11c, CD40, CD80, CD86, and MHC class II was highly upregulated (Figure 3B–F) and the proportions of CD40^+^, CD80^+^, CD86^+^, and MHC class II^+^ increased significantly (Figure S10B–E). These LPS-induced immunogenic responses were effectively suppressed by pretreatment with blank PDMAEMA-PLGA NPs, all doses of dexamethasone, and all doses of Dex-NPs. In addition, the immunosuppressive effect of blank NPs and Dex-NPs containing 1 μM and 2 μM dexamethasone on the expression of all immunogenic markers was comparable to the effect of 1 μM and 2 μM dexamethasone (Figure 3B–F and Figure S10A–E). 

The tolerogenic effects of PDMAEMA-PLGA NPs and Dex-NPs on the alteration of inhibitory molecules, ICOSL and PD-L1, were also observed (Figure 3G,H and Figure S10F,G). Upon LPS stimulation, the level of ICOSL expression was slightly downmodulated and the number of ICOSL^+^ cells was significantly decreased (Figure 3G and Figure S10F). Pretreatment with 1 μM dexamethasone slightly enhanced ICOSL expression and increased the proportion of ICOSL^+^ cells, but pretreatment with 2 μM dexamethasone decreased ICOSL expression and markedly reduced the proportion of ICOSL^+^ cells. Meanwhile, pretreatment with blank PDMAEMA-PLGA NPs could not alter ICOSL expression and decreased the proportion of ICOSL^+^ cells. Incorporation of dexamethasone into PDMAEMA-PLGA NPs neglected the suppressive activity of NPs in the ICOSL molecule and Dex-NPs containing dexamethasone less than 1 μM could maintain the expression of ICOSL, as well as the proportion of ICOSL^+^ cells (Figure 3G and Figure S10F). Unlike ICOSL expression, LPS-stimulated BM-cDCs showed highly upregulated PD-L1 expression and an increased proportion of PD-L1^+^ cells compared to unstimulated BM-cDCs (Figure 3H and Figure S10G). Pre-treatment with 1 μM and 2 μM dexamethasone downmodulated PD-L1 expression and rather reduced the proportion of PD-L1^+^ cells upon LPS stimulation. Consistently, blank PDMAEMA-PLGA NPs also exhibited suppressive activity on the PD-L1 molecule and incorporation of dexamethasone into NPs enhanced PD-L1 expression of PD-L1 compared to unstimulated BM-cDCs. Furthermore, pretreatment with Dex-NPs containing 1 μM and 2 μM dexamethasone could elevate PD-L1 expression in BM-cDCs to the same extent as LPS stimulation (Figure 3H). 

**Figure 4:**
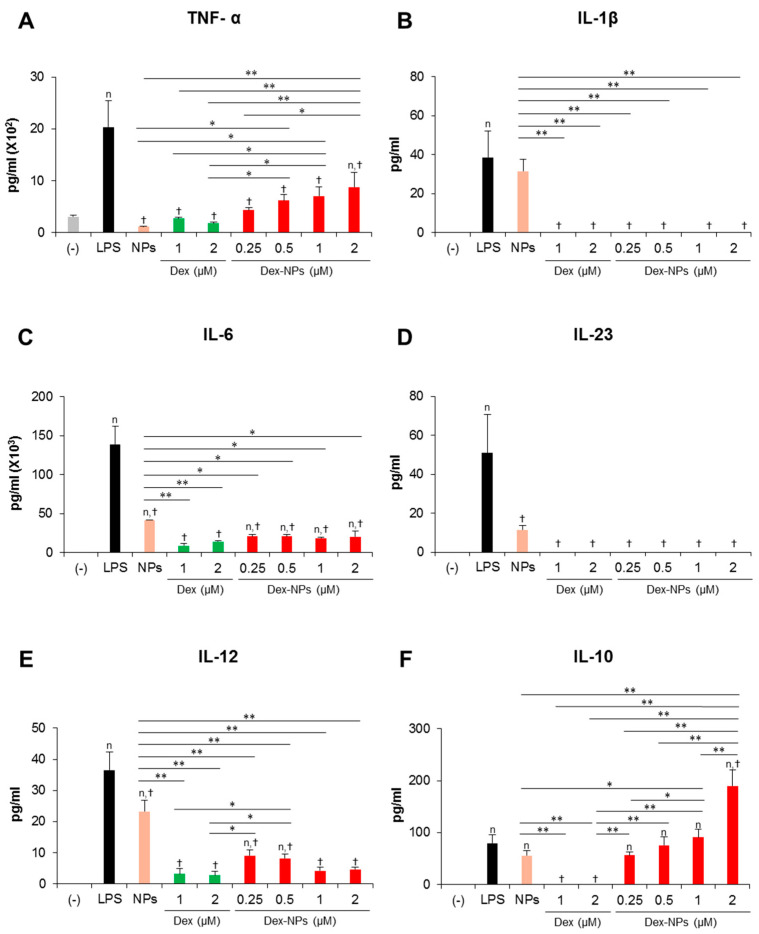
Effects of PDMAEMA-PLGA NPs and dexamethasone-incorporated PDMAEMA-PLGA NPs on the production of inflammatory and anti-inflammatory of wild-type BM-cDCs. Wild-type BM-cDCs (1 × 10^6^ cells/well) were untreated or pre-incubated with blank PDMAEMA-PLGA NPs (55 μg, an equal amount to Dex-NPs containing 2 μM dexamethasone), dexamethasone (1 and 2 μM), and dexamethasone-incorporated PDMAEMA-PLGA NPs containing 1 and 2 μM dexamethasone for 48 h. Subsequently, the DCs were stimulated with 0.1 μg/mL of LPS for 24 h. Culture supernatants were collected and (**A**) TNF-α, (**B**) IL-1β, (**C**) IL-6, (**D**) IL-23, (**E**) IL-12, and (**F**) IL-10 were determined using ELISA. *n* = 5; ^n^ *p* ≤ 0.05 compared with the negative control, ^†^ *p* ≤ 0.05 compared with LPS-stimulated BM-cDCs, * *p* ≤ 0.05, ** *p* ≤ 0.001; (−), negative control (untreated and unstimulated BM-cDCs); LPS, LPS-stimulated BM-cDCs; Dex, dexamethasone; Dex-NPs, dexamethasone-incorporated PDMAEMA-PLGA NPs; MFI, mean fluorescence intensity.

In parallel, the cytokine profiles of BM-cDCs were investigated (Figure 4). LPS stimulation enhanced the high levels of the inflammatory cytokines, TNF-α, IL-1β, IL-6, IL-23 and IL-12 (Figure 4A–E) and the moderate levels of the anti-inflammatory cytokine, IL-10 (Figure 4F). Pretreatment with blank PDMAEMA-PLGA NPs, all doses of dexamethasone, and all doses of Dex-NPs markedly decreased all inflammatory cytokine production in response to LPS stimulation. Blank NPs produced less suppressive effects on cytokine production compared to dexamethasone and Dex-NPs, while suppressive activities of Dex-NPs were comparable to dexamethasone (Figure 4A–E). Pretreatment with dexamethasone was unable to induce IL-10 production compared to LPS stimulation. Meanwhile, pretreatment with blank PDMAEMA-PLGA NPs and Dex-NPs containing 0.25–1 μM dexamethasone could induce IL-10 production to the same extent as LPS stimulation. Of interest, Dex-NPs containing 2 μM dexamethasone mediated the high IL-10 production compared to all treatments (Figure 4F). 

Collectively, our data demonstrated that PDMAEMA-PLGA NPs had immunosuppressive effects on cDC activation and inflammatory cytokine production, but did not have tolerogenic induction ability. Additionally, incorporation of dexamethasone into PDMAEMA-PLGA NPs improved the tolerogenic induction ability of both PDMAEMA-PLGA NPs and dexamethasone, in particular, the ability to induce ICOSL and PD-L1 expression and high IL-10 production.

### 2.4. PDMAEMA-PLGA NPs Altered the Hyperactivation of Fcgr2b^-/-^ BM-cDCs to Tolerogenic DCs

FCγRIIB is widely expressed on immune cells, including DCs and the mice deficient in FCγRIIB expression spontaneously developed lupus disease [24]. Additionally, mice with a specific deletion of *Fcgr2b* in DCs displayed enhanced immune responses and increased susceptibility to autoimmune diseases [25]. Our results also demonstrated that *Fcgr2b*^-/-^ BM-cDCs exhibited hyperactive phenotypes in response to LPS stimulation compared to WT BM-cDCs (Figure S11). 

**Figure 5:**
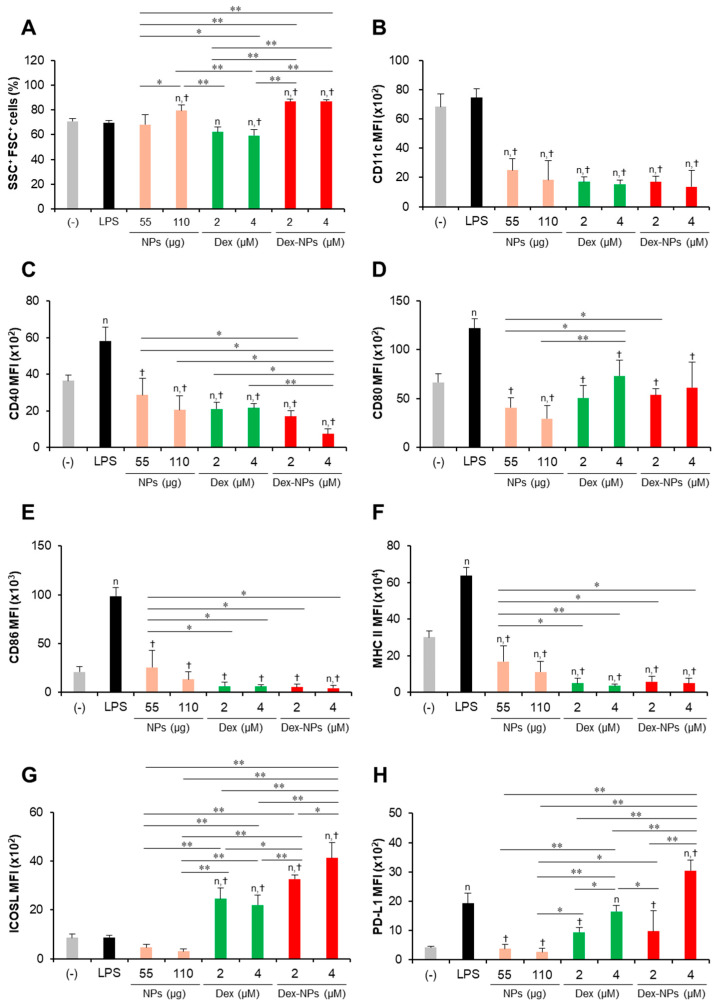
Immunosuppressive and tolerogenic effects of PDMAEMA-PLGA NPs and dexamethasone-incorporated PDMAEMA-PLGA NPs in *Fcgr2b*^-/-^ BM-cDCs. *Fcgr2b*^-/-^ BM-cDCs (1 × 10^6^ cells/well) were untreated or pre-incubated with blank PDMAEMA-PLGA NPs (55 μg and 110 μg, an equal amount to Dex-NPs containing 2 μM and 4 μM dexamethasone, respectively), dexamethasone (2 and 4 μM), and dexamethasone-incorporated PDMAEMA-PLGA NPs containing 2 and 4 μM dexamethasone for 48 h. Subsequently, the DCs were stimulated with 0.1 μg/mL of LPS for 24 h. (**A**) The percentages of SSC^+^FSC^+^ cells and the expression of (**B**) CD11c, (**C**) CD40, (**D**) CD80, (**E**) CD86, (**F**) MHC class II, (**G**) ICOSL, and (**H**) PD-L1 were evaluated by flow cytometry. *n* = 5; ^n^ *p* ≤ 0.05 compared with the negative control, ^†^ *p* ≤ 0.05 compared with LPS-stimulated BM-cDCs, * *p* ≤ 0.05, ** *p* ≤ 0.001; (−), negative control (untreated and unstimulated BM-cDCs); LPS, LPS-stimulated BM-cDCs; Dex, dexamethasone; Dex-NPs, dexamethasone-incorporated PDMAEMA-PLGA NPs; MFI, mean fluorescence intensity.

Therefore, we investigate whether our NPs are capable of suppressing the hyperactivation of *Fcgr2b*^-/-^ BM-cDCs and direct DCs to the tolerogenic phenotype. *Fcgr2b*^-/-^ BM-cDCs were pretreated with blank PDMAEMA-PLGA NPs, 2 and 4 μM dexamethasone, or Dex-NPs containing 2 and 4 μM dexamethasone and were subsequently stimulated with LPS. The DC marker, DC activation markers and DC tolerogenic markers were determined by flow cytometric analysis (Figure 5 and Figures S12–S18). All doses of dexamethasone reduced the percentages of SSC^+^ FSC^+^ cells in *Fcgr2b*^-/-^ BM-cDCs by approximately 8–10% when compared to the negative control, while LPS and 55 μg of the blank NPs did not affect this population. Interestingly, 110 μg of the blank NPs and all doses of Dex-NPs increased the percentages of SSC^+^ FSC^+^ cells (Figure 5A). Thus, dexamethasone, but not blank NPs and Dex-NPs, may affect cell viability as a result of the decrease in the percentages of SSC^+^ FSC^+^ cells. 

Consistent with the response of WT BM-cDCs (Figure 3 and Figure S9), blank PDMAEMA-PLGA NPs, dexamethasone, and Dex-NPs suppressed the expression of CD11c, CD40, CD80, CD86, and MHC class II to the same extent (Figure 5B–F and Figures S12–S14) and reduced the number of CD11c^+^, CD40^+^, CD80^+^, CD86^+^, and MHC class II^+^ (Figures S15–S18). Dexamethasone-incorporated NPs containing 4 μM dexamethasone exhibited the greatest ability to reduce the number of CD40^+^ and CD80^+^ cells and these results corresponded to those of blank PDMAEMA-PLGA NPs at 110 μg (Figure S18B,C).

LPS stimulation did not alter ICOSL expression, while it increased PD-L1 expression in *Fcgr2b*^-/-^ BM-cDCs (Figure 5G,H and Figure S17F,G) whose responses were similar to WT BM-cDCs (Figure 3G,H and Figure S9F,G). Consistently, blank PDMAEMA-PLGA NPs inhibited the expression of ICOSL and PD-L1. Of interest, all doses of Dex-NPs obviously enhanced ICOSL expression and increased the high number of ICOSL^+^ cells (Figure 5G and Figure S18F). Dexamethasone at 2 μM and 4 μM and Dex-NPs containing 2 μM maintained PD-L1 expression, while Dex-NPs containing 4 μM significantly enhanced PD-L1 expression and increased the high number of PD-L1^+^ cells (Figure 5H and Figure S18G) when compared to the negative control and LPS-stimulated *Fcgr2b*^-/-^ BM-cDCs.

**Figure 6:**
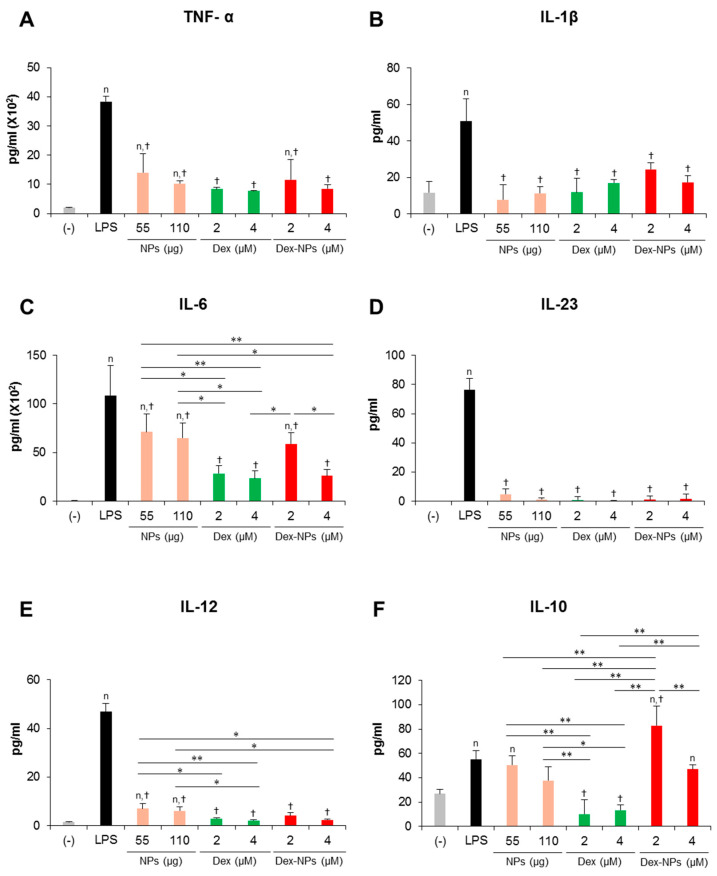
Effects of PDMAEMA-PLGA NPs and dexamethasone-incorporated PDMAEMA-PLGA NPs on the cytokine profiles of w *Fcgr2b*^-/-^ BM-cDCs. *Fcgr2b*^-/-^ BM-cDCs (1 × 10^6^ cells/well) were untreated or pre-incubated with blank PDMAEMA-PLGA NPs (55 μg and 110 μg, an equal amount to Dex-NPs containing 2 μM and 4 μM dexamethasone, respectively), dexamethasone (2 and 4 μM), and dexamethasone-incorporated PDMAEMA-PLGA NPs containing 2 and 4 μM dexamethasone for 48 h. Subsequently, the DCs were stimulated with 0.1 μg/mL of LPS for 24 h. Culture supernatants were collected and (**A**) TNF-α, (**B**) IL-1β, (**C**) IL-6, (**D**) IL-23, (**E**) IL-12, and (**F**) IL-10 were determined using ELISA. *n* = 5; ^n^ *p* ≤ 0.05 compared with the negative control, ^†^ *p* ≤ 0.05 compared with LPS-stimulated BM-cDCs, * *p* ≤ 0.05, ** *p* ≤ 0.001; (−), negative control (untreated and unstimulated BM-cDCs); LPS, LPS-stimulated BM-cDCs; Dex, dexamethasone; Dex-NPs, dexamethasone-incorporated PDMAEMA-PLGA NPs.

Inflammatory cytokine production in response to LPS stimulation was efficiently inhibited when *Fcgr2b*^-/-^ BM-cDCs were pretreated with blank PDMAEMA-PLGA NPs, dexamethasone, and Dex-NPs (Figure 6A–E). However, blank NPs partially suppressed IL-6 production (Figure 6C). Pretreatment with dexamethasone interfered with IL-10 production from *Fcgr2b*^-/-^ BM-cDCs in response to LPS stimulation. Meanwhile, IL-10 production from LPS-stimulated and blank PDMAEMA-PLGA NPs pretreated *Fcgr2b*^-/-^ BM-cDCs was not different. In particular, IL-10 production was further enhanced when *Fcgr2b*^-/-^ BM-cDCs were pretreated with Dex-NPs containing 2 μM, but not 4 μM, dexamethasone (Figure 6F).

Accordingly, blank PDMAEMA-PLGA NPs and Dex-NPs potentially suppressed the hyperactivation of *Fcgr2b*^-/-^ BM-cDCs by decreasing the expression of immunogenic markers and the production of inflammatory cytokines. Furthermore, Dex-NPs efficiently altered *Fcgr2b*^-/-^ BM-cDCs from immunogenic to tolerogenic phenotypes by enhancing the expression of ICOSL and PD-L1 and the production of IL-10. 

### 2.5. PDMAEMA-PLGA NPs Reinforced Dexamethasone to Promote Regulatory T Cell Expansion via BM-cDCs In Vitro

**Figure 7:**
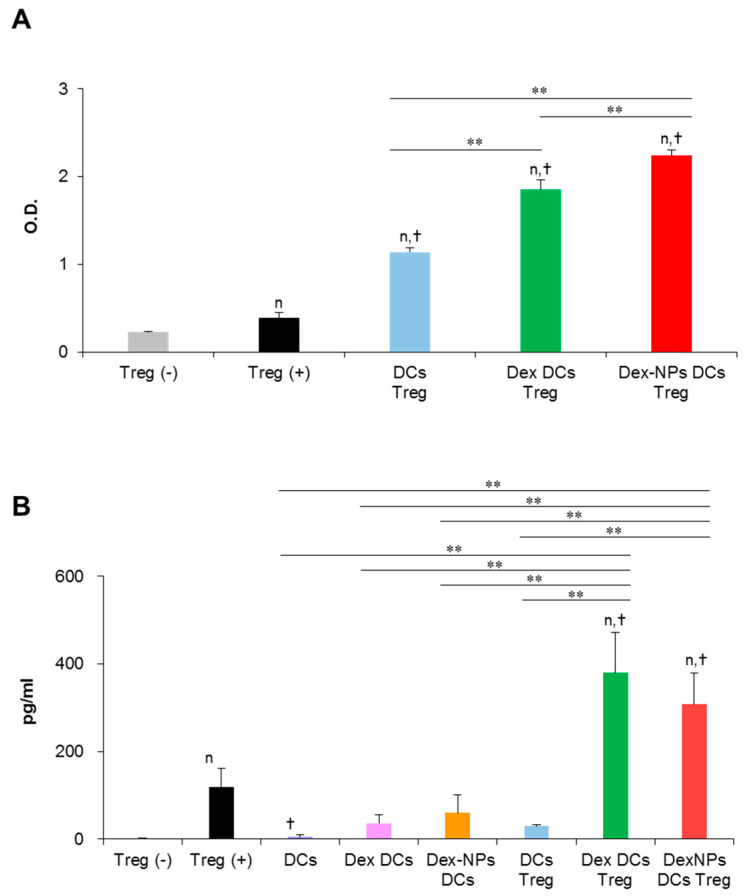
Direct in vitro interaction of dexamethasone-incorporated PDMAEMA-PLGA NPs pretreated BM-cDCs and regulatory T cells. Wild-type BM-cDCs were untreated or pre-incubated with 2 μM dexamethasone, or dexamethasone-incorporated PDMAEMA-PLGA NPs containing 2 μM dexamethasone for 48 h and BM-cDCs were stimulated with 0.1 μg/mL of LPS for 24 h. Subsequently, DCs were co-cultured with regulatory T cells isolated by magnetic-activated cell sorting in the presence of soluble anti-mouse CD3 mAb and recombinant mouse IL-2. (**A**) Cell proliferation was evaluated 72 h after co-culture and (**B**) IL-10 levels in the culture supernatant were measured 48 h after co-culture. *n* = 3; ^n^ *p* ≤ 0.05 compared with unstimulated regulatory T cells, ^†^ *p* ≤ 0.05 compared with regulatory T cells alone incubated with soluble anti-CD3 mAbs, ** *p* ≤ 0.001; Treg (−), unstimulated regulatory T cells; Treg (+), regulatory T cells incubated with soluble anti-mouse CD3 mAb and recombinant mouse IL-2; DCs Treg, LPS-stimulated BM-cDCs co-cultured with regulatory T cells; Dex-DCs Treg, dexamethasone pre-treated BM-cDCs co-cultured with regulatory T cells, Dex-NPs Treg, PDMAEMA-PLGA NPs pre-treated BM-cDCs co-cultured with regulatory T cells, DCs, LPS-stimulated DCs; Dex DCs, dexamethasone pre-treated BM-cDCs, Dex NPs DCs, dexamethasone-incorporated PDMAEMA-PLGA NPs pre-treated BM-cDCs.

A major property of tolerogenic DCs is to promote regulatory T cell (Treg) differentiation and function, thereby mediating tolerance. Therefore, we examined the ability of Dex-NPs pretreated BM-cDCs in regulatory T cell (Treg) proliferation and function in vitro (Figure 7). Since PDMAEMA-PLGA NPs improved the ability of dexamethasone to induce tolerogenic DCs (Figure 3, Figure 4, Figure 5 and Figure 6), in this experiment, we compared the function of tolerogenic DCs induced by Dex-NPs in comparison with dexamethasone. Dex-NPs pretreated BM-cDCs were pre-treated with 2 μM dexamethasone or Dex-NPs containing 2 μM dexamethasone and then stimulated with LPS. Subsequently, BM-cDCs were co-cultured with Tregs in the presence of soluble anti-CD3 mAb and recombinant IL-2 [26,27]. Seventy-two hours after co-cultures, the proliferation of Treg was measured by MTS assay. As a control for the system, regulatory T cells alone were cultured in the presence of soluble anti-CD3 mAb and recombinant IL-2 to ensure that the signals of soluble anti-CD3 mAb and recombinant IL-2 were optimal. BM-cDCs treated with dexamethasone and Dex-NPs enhanced Treg proliferation when compared to those of LPS-stimulated BM-cDCs. Furthermore, Dex-NPs promoted Treg expansion to a significantly higher extent than BM-cDCs pretreated with dexamethasone (Figure 7A).

A non-contact-dependent mechanism of Treg-mediated immune suppression is dependent on the production of IL-10 [28]. Thus, we evaluated Treg’s production of IL-10 in this in vitro DC/Treg co-culture system. Because IL-10 production was measured from the culture supernatant, it is possible that IL-10 may be derived from DCs that were co-cultured with Treg. Therefore, BM-cDCs stimulated with LPS and BM-cDCs pretreated with dexamethasone and Dex-NPs were cultured in parallel as controls. IL-10 production from these control BM-cDCs was very low, which means that IL-10 secreted from the DCs in the co-cultures did not interfere with the interpretation of the results. Control Treg alone incubated with soluble anti-CD3 mAbs and recombinant IL-2 also secreted a small amount of IL-10. Furthermore, LPS-stimulated BM-cDCs were unable to stimulate Treg to produce IL-10. BM-cDC were pretreated with dexamethasone and Dex-NPs potentially mediated Tregs to produce a substantial level of IL-10 to the same extent (Figure 7B).

### 2.6. PDMAEMA-PLGA NPs Were Actively Captured by Dendritic Cells In Vivo

**Figure 8:**
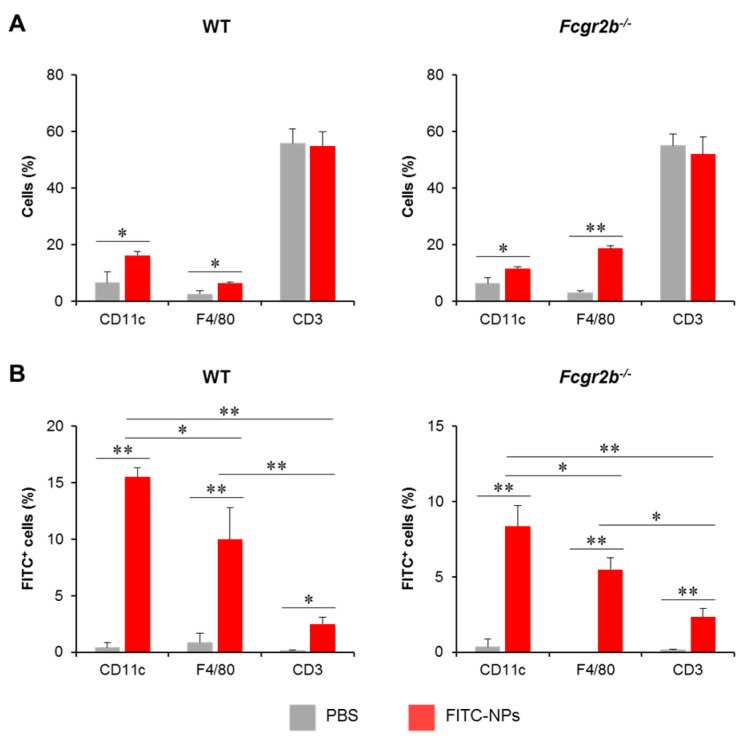
In vivo uptake of nanoparticles by dendritic cells. Phosphate buffer saline or FITC-tagged PDMAEMA-PLGA NPs were subcutaneously administered to wild-type and *Fcgr2b*^-/-^ mice. Seventy-two hours later, (**A**) The proportion of CD11c^+^, F4/80^+^, and CD3^+^ cells in the draining lymph nodes (dLNs) were determined by flow cytometric analysis. (**B**) The proportions of FITC^+^ cells in CD11c^+^, F4/80^+^, and CD3^+^ cells were evaluated by flow cytometric analysis. *n* = 5; * *p* ≤ 0.05, ** *p* ≤ 0.001; PBS, the control mice that received phosphate buffer saline; FITC-NPs, mice received FITC-tagged PDMAEMA-PLGA NPs.

To trace the in vivo uptake of PDMAEMA-PLGA NPs by DCs, PDMAEMA-PLGA NPs were tagged with FITC (FITC-NPs) and FITC-NPs were administered subcutaneously in WT and *Fcgr2b*^-/-^ mice. Three days later, FITC^+^ cells in skin draining LNs (dLNs) were analyzed along with DC, macrophage, and T cell markers which are CD11c, F4/80 and CD3, respectively (Figure S19). Wild-type and *Fcgr2b*^-/-^ mice received FITC-NPs showed significantly increased percentages of CD11c^+^ and F4/80^+^ cells, but not CD3^+^ cells, compared to PBS control mice (Figure 8A), indicating the migration of DCs and macrophages from the periphery to the draining LNs. 

To identify FITC^+^ cells in each immune cell population, sequential gating was placed on lineage markers (Figure S19A) and FITC^+^ cells (Figure S19B). In both WT and *Fcgr2b*^-/-^ mice, the number of FITC^+^CD11c^+^ cells increased while the number of FITC^+^F4/80^+^ cells increased moderately. Although the population was detectable, the numbers of this population were considerably smaller than the number of FITC^+^CD11c^+^ cells (Figure 8B). These results indicated that the NPs were captured and transported by DCs and, to a lesser extent, macrophages. In addition, we traced the distribution of FITC-NPs in the non-T, non-DC, and non-macrophage population (Figure S20). These non-lineage cells were identified by sequential gating strategies (Figure S20A) and FITC^+^ cells were then analyzed (Figure S20B). The FITC^+^ cells in this population were less than 2% (Figure S20C), supporting that FITC-NPs were mainly captured by DCs (Figure 8B). 

### 2.7. Dexamethasone-Incorporated PDMAEMA-PLGA NPs Alleviated Lupus Disease in Fcgr2b^-/-^ Mice

**Figure 9:**
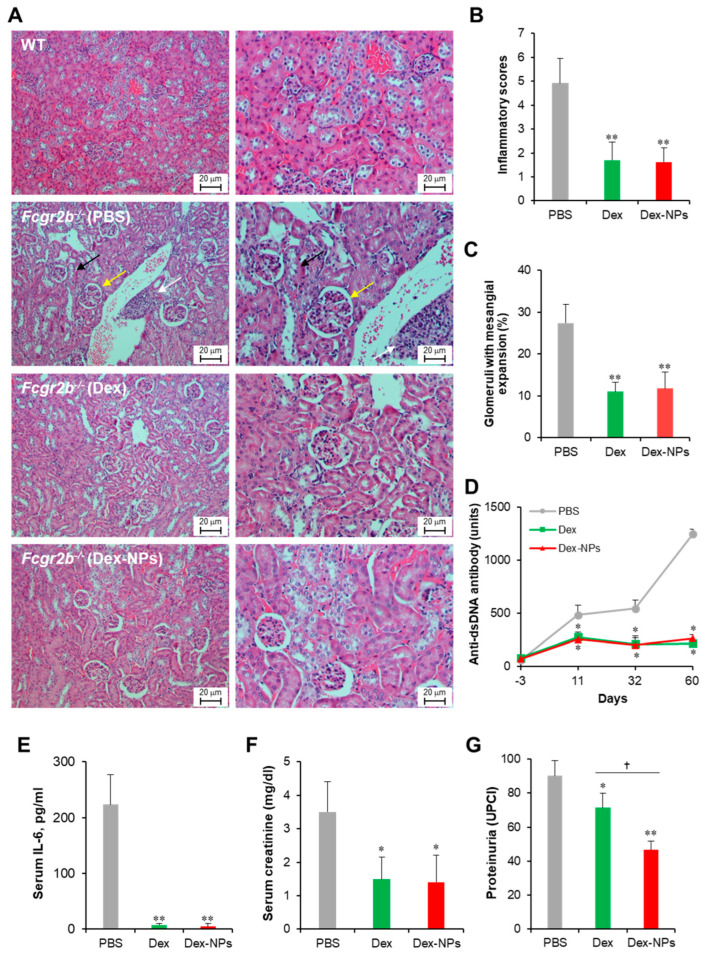
Potent amelioration of lupus disease by dexamethasone-incorporated NPs. Renal damages of wild-type (WT) and *Fcgr2b*^-/-^ mice treated with phosphate buffer solution (PBS) and apoptotic bodies mixed with dexamethasone (Dex) or dexamethasone-incorporated PDMAEMA-PLGA NPs (Dex-NPs) were indicated by (**A**) Representative of histopathology with H–E staining, (**B**) renal tubulointerstitial injury score, and (**C**) glomerular injury are demonstrated. Serum was also collected from all mice to investigate the alteration of (**D**) anti-double strand DNA auto-antibodies, (**E**) IL-6, (**F**) creatinine, and (**G**) Proteinuria was measured and represented in the urine protein creatinine index (UPCI). *n* = 5; * *p* ≤ 0.05 compared with PBS control, ** *p* ≤ 0.001 compared with PBS control, ^†^ *p* ≤ 0.05. The arrows are indicated for the representative lesions as the follows; black arrow, tubular vacuolization; yellow arrow, glomeruli with mesangial expansion; white arrow, interstitial infiltration.

Next, we compared the therapeutic potential of DC-targeted delivery using Dex-NPs in comparison with the non-targeted dexamethasone therapy in a lupus mouse model. Apoptotic bodies are recognized as auto-antigens in SLE and are critical factors that aggravate the immunopathogenesis of SLE [29]. Therefore, we used apoptotic bodies from *Fcgr2b*^-/-^ mice that exhibited lupus disease as self-antigens to induce adaptive immune responses. *Fcgr2b*^-/-^ mice with onset of lupus (16–24 weeks old) were subcutaneously injected with PBS, or self-antigens (apoptotic bodies) mixed with dexamethasone or Dex-NPs weekly for four consecutive weeks and alteration of kidney histopathology, serum anti-dsDNA antibodies, serum IL-6, serum creatinine and urine protein/creatinine was observed (Figure 9). Control *Fcgr2b*^-/-^ mice that received PBS apparently showed lupus nephritis as indicated by renal tubulointerstitial injury scores based on tubular vacuolization and increased infiltration of interstitial cells (Figure 9A, second row, and Figure 9B). Similarly, the prominent glomerular lesion of lupus nephritis, a pathognomonic lesion of the disease indicated by the percentage of glomeruli with moderate to severe mesangial expansion, was detectable in *Fcgr2b*^-/-^ control mice (Figure 9A, second row and Figure 9B). Of interest, *Fcgr2b*^-/-^ lupus-prone mice treated with dexamethasone or Dex-NPs exhibited less severe renal interstitial inflammation and lower glomeruli with mesangial expansion (Figure 9A, third and fourth rows, and Figure 9B,C). Consistently, anti-dsDNA antibodies, serum IL-6, and serum creatinine levels were substantially decreased in mice treated with dexamethasone or Dex-NPs (Figure 9D–F). In addition, dexamethasone and Dex-NP treatment significantly reduced proteinuria (urine protein creatinine index; UPCI) in *Fcgr2b*^-/-^ lupus-prone mice (Figure 9G). As a result, DC-targeted therapy using Dex-NP improved lupus outcomes and its therapeutic effects were comparable to those of dexamethasone. 

### 2.8. PDMAEMA-PLGA NPs Incorporated with Dexamethasone Upregulated the Expression of ICOSL and PD-L1 in DCs and Enhanced Regulatory T Cell Expansion in Fcgr2b^-/-^ Lupus-Prone Mice

**Figure 10:**
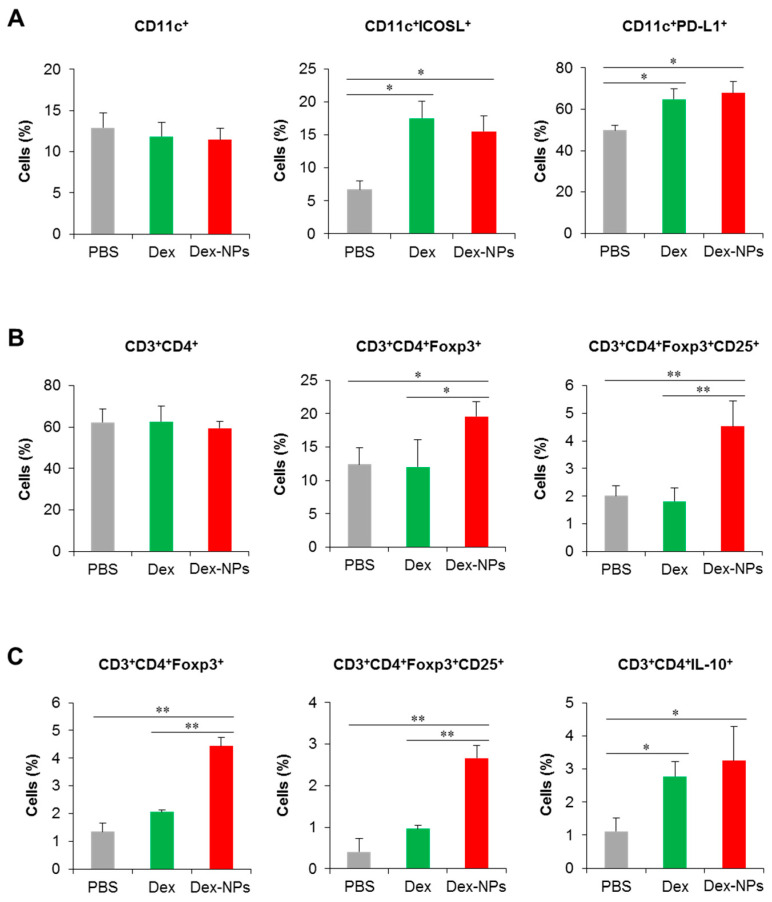
Investigation of the DC and T cell population in lupus mice and in vitro restimulation of draining lymph node cells. *Fcgr2b*^-/-^ mice were treated with phosphate buffer solution (PBS) and apoptotic bodies mixed with dexamethasone (Dex) or dexamethasone-incorporated PDMAEMA-PLGA NPs (Dex-NPs). At the end point of treatment, dLNs were collected and (**A**) tolerogenic DC phenotypes and (**B**) the CD4 T cell and Treg population were determined by flow cytometric analysis. (**C**) LN cells were restimulated with apoptotic bodies in vitro and the Treg population was evaluated. *n* = 5; * *p* ≤ 0.05, ** *p* ≤ 0.001.

To further validate the superior therapeutic efficacy of DC-targeted therapy using dex-NPs, the alterations of DC phenotypes and the Treg population were investigated. *Fcgr2b*^-/-^ lupus-prone mice were administered with PBS and the antigens mixed with dexamethasone or Dex-NPs as described above and the dLNs were collected for the analysis of DCs and T cells by flow cytometry (Figure S21). Compared to PBS control mice, administration of dexamethasone and Dex-NPs did not alter the proportion of CD11c^+^ cells (Figure 10A, right panel), but both treatments significantly increased the number of CD11c^+^ICOSL^+^ and CD11c^+^PD-L1^+^ cells (Figure 10A, middle and left panel), indicating the successful induction of tolerogenic DCs in situ. The numbers of CD3^+^CD4^+^ cells were also not affected by treatment with dexamethasone and Dex-NP (Figure 10B, right panel). Of interest, Dex-NPs, but not dexamethasone, mediated the expansion of CD3^+^CD4^+^Foxp3^+^ Tregs and CD3^+^CD4^+^Foxp3^+^CD25^+^ activated Tregs in *Fcgr2b*^-/-^ lupus-prone mice (Figure 10B, middle and left panel). 

Parallelly, we performed the in vitro restimulation assay to determine antigen-specific responses. LN cells from the dLNs of *Fcgr2b*^-/-^ lupus-prone mice treated with PBS, dexamethasone and Dex-NPs as described above were restimulated with apoptotic bodies and Treg populations were evaluated by flow cytometric analysis. Consistent with in vivo observation, only Dex-NP treatment enhanced the number of CD3^+^CD4^+^Foxp3^+^ and CD3^+^CD4^+^Foxp3^+^CD25^+^ cells in response to re-exposure of a specific antigen (Figure 10C, right and middle panel). In this experiment, we also observed the production of IL-10 by CD4^+^ T cells and found that dexamethasone and Dex-NP treatment showed a similarly elevated level of the CD3^+^CD4^+^IL-10^+^ population (Figure 10C, left panel). Collectively, the data indicated that targeting DCs in situ using Dex-NPs reprogramed DCs to a tolerogenic phenotype and consequently mediated the antigen-specific Treg response.

## 3. Discussion

In the drug release assay, PDMAEMA-PLGA NPs demonstrated the stable and sustained drug release profiles. Previous observations in dexamethasone-incorporated PLGA NPs showed that the release rate of the in vitro system ranged between 5–60% [30,31]. Although there is no direct evidence to indicate the release of dexamethasone from PDMAEMA NPs, an in vitro study demonstrated the effective control release of PDMAEMA NP (10–50%) [32]. In collaboration with our results, the copolymer of PDMAEMA and PLGA provided a sustained release profile, although the drug was adsorbed onto the surface of the NPs. This slow release rate of Dex-NPs may be explained by the binding of dexamethasone to the NPs via electrostatic interactions (Figure 1 and Table 1).

Unlike previous work that often used GM-CSF and IL-4-derived BM-DCs as the primary DC platform for the in vitro study, this work used BM-cDCs derived from FLT3L and GM-CSF. Evidence demonstrated that BM-DCs derived from GM-CSF with or without IL-4 comprise a heterogeneous population of myeloid cells such as DCs and macrophages [33]. Therefore, BM-DCs derived from GM-CSF and IL-4 may not represent conventional DCs. In contrast, FLT3L-derived BM-DCs composed of a homogeneous population of cDCs and the addition of GM-CSF gave rise to DCs as the dominant phenotypes of cDC1 and cDC2 [21,22,23]. With this cDC culture system, more insight in DC immunity will be provided. 

In our in vitro primary cDC platform, dexamethasone could still inhibit activation and inflammatory responses (Figure 3B–F and Figure 4A–E), which is similar to GM-CSF-derived BM-DCs (with or without IL-4) pretreated with dexamethasone [34,35,36]. Although there is no direct evidence for the effect of dexamethasone on the expression of ICOSL and PD-L1 in DC, a few reports in other cell types demonstrated down-modulation of ICOSL and PD-L1 by dexamethasone [27,37]. Our data also showed that dexamethasone interfered with the expression of PD-L1 (all doses of dexamethasone) and ICOSL expression (2 μM dexamethasone) (Figure 3G,H). Previously, dexamethasone-incorporated PLGA NPs were found to be unable to induce PD-L1 expression in DCs [38], but our PDMAEMA-PLGA NPs produced a higher result in the induction of PD-L1 expression (Figure 3H). 

High IL-10 production was detected in GM-CSF-derived BM-DCs treated with dexamethasone [35,36]; however, dexamethasone was unable to induce IL-10 production in the BM-cDC system (Figure 4F), indicating the different characteristics of both DCs. Unlike dexamethasone, PDMAEMA-PLGA NPs had the ability to induce IL-10 production in BM-cDCs and PDMAEMA-PLGA NPs further promoted dexamethasone to produce high IL-10 (Figure 4F). A previous report demonstrated that IL-10 production in vivo increased by treatment with a low dose of dexamethasone. Therefore, it is likely that the slow release of drugs controlled by PDMAEMA-PLGA NPs may lead to a lower dose of dexamethasone, which consequently affected enhanced IL-10 production in BM-cDCs. 

Hyperresponsiveness and dysfunction of DCs in SLE promote and perpetuate the activation of autoreactive T cells, which consequently contributes to the progression and aggravation of the disease [29]. PDMAEMA-PLGA NPs could substantially suppress the hyperactive *FcgR2b*^-/-^ BM-cDCs, and PDMAEMA-PLGA NPs could still improve the tolerogenic induction properties of dexamethasone in the *FcgR2b*^-/-^ system (Figure 3, Figure 4, Figure 5 and Figure 6). Therefore, this PDMAEMA-PLGA NP delivery system may offer an alternative strategy for modulating aberrant DC functions. 

The physicochemical properties of NPs can affect the immune outcome of DCs by altering their maturation and function [39]. Our previous in vitro studies also verified the effects of PLGA NPs and PDMAEMA NPs on DC functions. PLGA NPs induced immunogenic responses in BM-DCs, as DCs increased the expression of their activation markers and increased the production of inflammatory cytokines [11]. PLGA NPs also showed good immunogenicity in vivo since they were able to activate DCs and the adaptive immune responses in mice [40,41,42]. In contrast, PDMAEMA NPs inhibited the activation of BM-DCs and interfered with inflammatory cytokines in DCs [10]. Therefore, the immunomodulatory capabilities of PDMAEMA-PLGA NPs in this study possibly resulted from the binding of PDMAEMA to the surface layer of the nanocomposites, as in the proposed nanostructure (Figure 1 and Table 1). 

Evidences have demonstrated the role of ICOSL, PD-L1, and IL-10 in Treg differentiation and function [1,43]. DCs expressing ICOSL and IL-10 induced Treg expansion with potent inhibitory function through high IL-10 production [44]. PD-L1 expression in DC is required for the development and immunosuppressive function of Treg [1,45]. Although BM-cDCs pretreated with dexamethasone did not produce IL-10 (Figure 4F), the coexpression of ICOSL and PD-L1 in company with immature phenotypes (low expression of immunogenic molecules) (Figure 3C–H) may be these mechanisms that lead to expansion and IL-10 production of Treg [46,47]. The pretreated BM-cDCs with Dex-NPs also exhibited the immature phenotype, but the DCs expressed ICOSL and high IL-10 PD-L1 along with high production (Figure 4F–H) which possibly resulted in Treg expansion and IL-10 production (Figure 7). It is noteworthy that Dex-NP pretreated BM-cDCs could mediate Treg expansion better than dexamethasone pretreated BM-cDCs (Figure 7A). This result may be due to the difference in IL-10 production induced by dexamethasone and Dex-NPs (Figure 4F) since induction of Treg differentiation by DCs required IL-10 signaling [48,49].

DCs have been elucidated to favor polymeric nanoparticles with sizes ranging from 100 to 600 nm [39]. We previously compared the in vitro uptake of DCs in response to 300 nm and 500 nm PLGA NPs and found that 500 nm NPs had a higher uptake efficiency by DCs [11]. Thus, in this study, we manufactured PDMAEMA-PLGA NPs with a size of approximately 500 nm (Figure 1A). As expected, NPs were actively captured and transported by DCs and, to a lesser extent, macrophages in WT and *Fcgr2b*^-/-^ mice (Figure 8B). A study investigating in vivo trafficking of NPs of various sizes revealed that skin DCs were strictly required to transport polymeric NPs of 500–2000 nm from the skin to the dLNs [16]. Furthermore, macrophages, with less capability, could uptake NPs of similar size [16]. Therefore, in vivo uptake of PDMAEMA-PLGA NPs was possibly mediated by the size-dependent manner.

In this study, we aimed to induce tolerogenic DCs in situ using immunomodulatory NPs that incorporate dexamethasone for the treatment of SLE. Since DCs are a major population that resides in the skin, NP administration via the subcutaneous route may provide precise delivery to DCs. Our data also suggested that PDMAEMA-PLGA NPs had a high efficiency to enter skin DCs (Figure 8); therefore, Dex-NP delivery in vivo may specifically target DCs. Our findings demonstrated that DC-targeted therapy using PDMAEMA-PLGA NPs produced favorable therapeutic effects in the lupus-prone mouse model as same as the non-targeted therapy with dexamethasone (Figure 9). 

In this study, our aim was to target DCs in situ and harness them using immunomodulatory NPs for the treatment of SLE. Since DCs are a major population that resides in the skin, NP administration via the subcutaneous route may provide precise delivery to DCs. Our data also suggested that PDMAEMA-PLGA NPs had a high efficiency to enter skin DCs (Figure 8); therefore, Dex-NP delivery in vivo may specifically target DCs. Our findings demonstrated that DC-targeted therapy using PDMAEMA-PLGA NPs produced favorable therapeutic effects in the lupus-prone mouse model as comparable as the non-targeted therapy with dexamethasone (Figure 9). It was surprising that our DC-targeted therapy using the PDMAEMA-PLGA NP system produced similar in vivo therapeutic effects to the non-targeted dexamethasone therapy that broadly affected several cells, including other immune cells and parenchymal cells in several organs. These data suggested the importance of innate immune cells, especially DCs, in lupus disease, which is a disease of aberrant functions of innate and adaptive immune cells [3]. In addition, specific interference of DC functions can control the fate of adaptive immune responses through T helper cells and potentially attenuated lupus nephritis (reduced anti-dsDNA, proteinuria, and renal injury). Therefore, in situ DC-targeted therapy may be a better alternative approach for the treatment of SLE and other autoimmune diseases.

The in vitro restimulation assay substantiated that the increased FoxP3^+^ Tregs in lupus mice treated with Dex-NPs were induced in an antigen-specific manner and this Treg expansion was probably impelled by DCs since the direct in vitro interaction between the BM-cDCs pretreated with Dex-NPs and Treg led to substantial Treg expansion (Figure 7A). Observation of the tolerogenic phenotypes of DCs in *Fcgr2b*^-/-^ mice revealed similar abilities of dexamethasone and Dex-NPs in the induction of ICOSL and PD-L1 expression (Figure 10A); however, the outcomes of Treg responses were different (Figure 10B,C). The substantial number of Tregs in Dex-NPs treated lupus mice may primarily depend on IL-10 production by DCs since Dex-NPs, but not dexamethasone, induced high IL-10 production in both WT and *Fcgr2b*^-/-^ DCs (Figure 4F and Figure 6F). As mentioned above, IL-10 production by DCs is essential for the development and expansion of FoxP3^+^ Tregs [39]. 

Since the balance between immune effectors and regulators is critical for immune homeostasis and the control of autoimmunity, either exacerbated effector responses or insufficient immune regulation can lead to inflammatory diseases and autoimmune disorders. Thus, approaches to increasing the number and function of Treg cells can provide great benefit to patients with autoimmune disorders [28]. Tolerogenic DCs play a key role in the promotion of central and peripheral tolerance through various mechanisms, including Treg induction [2]. The use of the PDMAEMA-PLGA NP system possibly allowed the precise targeting to DCs (Figure 8) and enabled the tolerogenic induction in situ (Figure 1). In addition, this selective targeting to DCs mediated antigen-specific immune tolerance via the induction of Treg expansion (Figure 10B,C) and consequently led to a potent amelioration of lupus disease (Figure 9). The future investigation of the bioavailability and systemic release of Dex-NPs will provide additional insight into the clinical utilization of this nanoparticle platform for the treatment of SLE and other autoimmune diseases.

## 4. Materials and Methods

### 4.1. Preparation of PDMAEMA-PLGA Nanoparticles

PDMAEMA-PLGA NPs were synthesized following our previous publications with modifications using a single emulsion/solvent evaporation method [9,10,11]. One hundred milligrams of poly(lactic-co-glycolic acid) with lactide:glycolide of 50:50 (Sigma, St. Louis, MO, USA) were dissolved in 10 mL of dichloromethane (RCI Labscan, Bangkok, Thailand). The aqueous solution was prepared by dissolving 10 mg of PDMAEMA (MW 10 kDa) that was synthesized following our previous publication [9] and 120 mg of polyvinyl alcohol (PVA, MW 9–10 kDa, 80% hydrolyzed; Sigma) in 30 mL of endotoxin-free water. The organic solution of PLGA was then homogenized in the prepared aqueous solution using a handheld homogenizer (ErgoMixx, Bosch, Gerlingen, Germany) for 2 min. Subsequently, the organic solvent was evaporated at 25 °C under magnetic stirring for 2 h. The obtained particles were centrifuged at 500 rcf to remove large particles with the size greater than 1000 nm. The supernatant was then centrifuged at 2000 rcf to collect NPs with 500 nm in size. PLGA-PDMAEMA NPs were lyophilized and stored at 4 °C until use.

### 4.2. Preparation of Dexamethasone-Incorporated Nanoparticles

Dexamethasone was incorporated into the nanoparticles by mixing the dexamethasone solution (2 mg of dexamethasone (Sigma-Aldrich, St. Louis, MO, USA) in 1 mL of methanol) with the suspension of PLGA-PDMAEMA NPs (10 mg of NPs in 8 mL of water) under magnetic stirring for 1 h. Subsequently, the suspension was centrifuged at 2500 rcf and the supernatant was collected for the measurement of free dexamethasone and the dexamethasone-incorporated NPs were collected and redispersed in water (at a concentration of 10 mg/mL) and stored at 4 °C. 

To measure the amounts of dexamethasone that was incorporated into the NPs, the free dexamethasone in the supernatant was measured with a UV-VIS spectrophotometer at absorbance of 242 nm and 600 nm [20]. The buffer (1:8 of methanol:water) was used as a baseline control. The amounts of free dexamethasone were calculated using the following formula: (O.D. 242 nm − O.D. 600 nm) × Extinction coefficient (13.770 × 10^3^/M^−1^/cm^−1^) [20]. To calculate the amounts of incorporated dexamethasone, the initial amounts of dexamethasone (2 mg) were subtracted with the amounts of free dexamethasone.

### 4.3. Preparation of FITC-Tagged Nanoparticles

Fluorescein isothiocyanate (FITC, >90%, Sigma-Aldrich) was dissolved in ethanol at a concentration of 500 µg/mL, and 20 mg of PDMAEMA-PLGA NPs was dispersed in 800 µL endotoxin-free water. Then, eighty microliters of the FITC solution was added to the PDMAEMA-PLGA nanoparticle suspension. The mixture of FITC and NPs was stirred for 1 h at room temperature and then kept at 4 °C for 24 h. Subsequently, the mixture was mixed by vortex and repeatedly washed with endotoxin-free water until the fluorescence disappeared from the supernatant. All procedures were performed in the dark to protect the FITC from light. FITC-tagged NPs were dispersed in endotoxin-free water and stored in the dark at 4 °C.

### 4.4. Material Characterization

The morphology and size of PLGA NPs and PDMAEMA-PLGA NPs were determined using a scanning electron microscope (SEM) (JSM-IT100, JEOL, Tokyo, Japan) after sputtering coated with gold. Thermogravimetric analysis (TGA) and differential thermogravimetric analysis (DTG) were carried out with the TG 209F3 instrument (NETZSCH, Selb, Germany). Before each measurement, all samples were lyophilized to remove most of the water and volatile solvent. All samples were heated from 50 to 600 °C with a heating rate of 20 °C/min under a nitrogen atmosphere.

The hydrodynamic diameter, polydispersity indices and charges of PLGA NPs, PDMAEMA-PLGA NPs and dexamethasone-incorporated PDMAEMA-PLGA NPs were measured using a dynamic light scattering and zeta potential analyzer (ZSP; Zetasizer, Malvern Instruments Ltd., Worcestershire, UK). All measurements were made in Milli-Q water.

### 4.5. Drug Release Assay

PDMAEMA-PLGA NPs and dexamethasone-incorporated PDMAEMA-PLGA NPs at a concentration of 1 mg per 2 mL water were incubated at 4 °C and 37 °C in dark without stirring and shaking. On days 0, 7, 14, 21, and 28, the NPs were centrifuged at 2500 rcf and the supernatants were collected for the measurement of free dexamethasone. The free dexamethasone in the supernatants was determined at absorbances of 242 nm and 600 nm. The amounts of free dexamethasone were calculated using the following formula: [(O.D. 242 nm − O.D. 600 nm) of Dex-NPs − (O.D. 242 nm − O.D. 600 nm) of blank NPs] × Extinction coefficient (13.770 × 10^3^/M^−1^/cm^−1^) [20]. The percentages of drug release were calculated by normalizing the amounts of free dexamethasone each day to day 0.

### 4.6. Animals and Ethical Statement

*Fcgr2b*^-/-^ mice (C57BL/6 background) were obtained from Dr. Silvia Bolland (NIAID, NIH, Bethesda, MD, USA) [24]. C57BL/6 mice were purchased from Nomura Siam International Co, Ltd., Bangkok, Thailand. All animals were kept and housed at the Animal Facility Center of the Faculty of Medicine, Chulalongkorn University. Mice at 5–6 weeks of age were used for the generation of BMDC in vitro and mice at 4–6 months of age were used for in vivo experiments. 

### 4.7. Generation of BM-cDCs 

Bone marrow-derived cDCs were generated following previous publications [22,23]. Briefly, bone marrow cells were collected from mouse femurs and tibias. Cells were seeded in a 24 well plate at a concentration of 1 × 10^6^ cells/mL, and cultured in RPMI 1640 medium (GIBCO, Thermo Fisher Scientific, Rochester, NY, USA) supplemented with 10% heat-inactivated fetal bovine serum (HI-FBS, GIBCO), 2 mM Glutamax (GIBCO), penicillin (100 IU/mL) and streptomycin (100 ug/mL) (GIBCO), and recombinant mouse FLT3L (200 ng/mL) (BioLegend, San Diego, CA, USA). Half of the culture medium was replaced on day 3 and day 6. Recombinant mouse GM-CSF (20 ng/mL) was added to the culture on day 6 and day 8. Cells were incubated at 37 °C, 5% CO_2_ in a humidified atmosphere. 

### 4.8. In Vitro Determination of BM-cDC Activation

On day 7 of the BM-cDC culture, WT BM-cDCs were treated with 1 and 2 μM dexamethasone, dexamethasone-incorporated NPs containing 0.25, 0.5, 1 and 2 μM of dexamethasone, and 110 μg unincorporated NPs (equal to the amount of Dex-NPs containing 2 μM of dexamethasone), and *Fcgr2b*^-/-^ BM-cDCs, cells were treated with 2 and 4 μM dexamethasone, dexamethasone-incorporated NPs containing 2 and 4 μM of dexamethasone, and 55 and 110 μg unincorporated NPs (equal to the amount of Dex-NPs). On day 9 of culture, cells were stimulated with LPS (0.1 μg/mL, Sigma) for 24 h. On day 10, the culture supernatants were harvested for cytokine quantification and the cells were collected for flow cytometric analysis. The negative control was untreated and unstimulated BM-cDCs, and the positive control was LPS-stimulated BM-cDCs.

The surface markers, CD11c, CD40, CD80, CD86, MHC class II, PD-L1, and ICOSL, were determined by flow cytometric analysis. The production of cytokines, TNF-α, IL-1β, IL-6, IL-23, IL-12 and IL-10 were assessed by ELISA.

### 4.9. In Vitro Co-Culture of BM-cDCs and Regulatory T Cells

The spleens and LN of WT mice were collected and digested with 300 units/mL collagenase IV (Invitrogen/ThermoFisher Scientific, Rochester, NY, USA) and 10 units/mL DNase I (Invitrogen/ThermoFisher Scientific) at 37 °C shaking at 150 rpm for 45 min. Subsequently, the cells were washed and the Tregs were sorted by immunomagnetic beads (CD4^+^CD25^+^ Regulatory T Cell Isolation Kit, mouse; Miltenyi Biotec, San Jose, CA, USA). The purity of the sorted Tregs was evaluated by flow cytometric analysis (>80%). 

Bone marrow-derived cDCs were untreated, treated with 2 μM dexamethasone, or Dex-NPs containing 2 μM of dexamethasone for 48 h as described above and stimulated with 0.5 μg/mL LPS for 24 h. BM-cDCs were washed 3 times and co-cultured with Treg in a Treg:DC ratio of 2:1 in RPMI1640 supplemented with 10% HI-FBS, 2 mM Glutamax, penicillin (100 IU/mL) and streptomycin (100 μg/mL), and 2-Mercaptoethanol (55 μM, GIBCO). 

Treg/DC was co-cultured in the presence of 500 ng/mL soluble anti-mouse CD3 mAbs (145-2C11, Biolegend), and 5 ng/mL recombinant mouse IL-2 (Biolegend) in 96 well plates for IL-10 detection [26,27], and Treg/DC were co-cultured in the presence of 50 mg/mL anti-mouse CD3 mAbs and 5 ng/mL recombinant mouse IL-2 for the Treg proliferation assay [26,27]. For control, Tregs alone were cultured with or without soluble anti-CD3 mAbs and IL-2. 

Culture supernatants were collected at 48 h for measurement of IL-10 production by ELISA. Treg proliferation was determined at 72 h using the CellTiter 96^®^ Aqueous One Solution Cell Proliferation Assay (MTS, Promega, Madison, WI, USA) following the manufacturer’s instructions. 

### 4.10. In Vivo Uptake of FITC-NPs

Mice were administered subcutaneously with FITC-incorporated NPs (1 mg in 100 µL PBS) on the neck scuff. Control mice received 100 µL PBS. Seventy-two hours later, the draining LNs (cervical and brachial LNs) were collected and the LN cells were isolated by collagenase/DNase I digestion as described above. FITC^+^ cells and lineage markers, CD11c, F4/80, and CD3, were determined by flow cytometric analysis.

### 4.11. Preparation of Apoptotic Bodies

Apoptotic bodies were prepared following a previous publication [50]. Briefly, spleens were collected from the ages of *Fcgr2b*^-/-^ mice that exhibited lupus disease. The splenocytes (2 × 10^6^ cells/mL) were treated with 1 μM of staurosporine for 24 h. The treated splenocytes were centrifuged at 300 g at room temperature for 5 min. The supernatant was collected and subsequently centrifuged at 2000 g at room temperature for 20 min. After centrifugation, the supernatant was discarded and the pellet of the apoptotic bodies was collected and resuspended in PBS. The amounts of apoptotic bodies were determined by the Bradford protein assay. 

### 4.12. In Vivo Drug Delivery

*Fcgr2b*^-/-^ mice (16–20 weeks of age with lupus onset [51]) were used for the in vivo drug delivery. Mice were subcutaneously administrated with 100 μg of dexamethasone or dexamethasone-incorporated NPs containing 100 μg of dexamethasone together with 25 μg of apoptotic bodies in PBS weekly for 4 consecutive weeks (days 0, 7, 14, and 21). Negative control mice subcutaneously received 200 μL PBS. 

The sera were collected 3 days before the first administration and on days 11, 32, and 60). On day 60, the kidneys were harvested for histopathological examination and dLNs were harvested for the assessment of the DCs (CD11c, ICOSL, PD-L1) and T cells (CD3, CD4, CD25, Foxp3, and IL-10) by flow cytometry.

### 4.13. In Vitro Restimulation Assay

dLN cells and splenocytes (2 × 10^6^ cells/mL) from the in vivo drug delivery experiment were restimulated with 20 μg/mL of apoptotic bodies for 72 h. Subsequently, the Treg population was determined using markers, CD3, CD4, CD25, Foxp3, and IL-10, by flow cytometry.

### 4.14. Flow Cytometric Analysis

Fluorochrome conjugated monoclonal antibodies against mouse CD11c (N418), CD40 (3/23), CD80 (16-10A1), CD86 (GL-1), MHC class II (M5/114.15.2), ICOSL (HK 5.3), PD-L1 (10F.9G2), CD3 (145-2C11), CD4 (GK1.5), CD25 (PC61), Foxp3 (FK-16s) and IL-10 (JES5-16E3) were obtained from Biolegend. 

For cell surface staining, cells were resuspended in a staining buffer (1% FBS and 0.1% sodium azide in 1X PBS) and blocked with purified anti-mouse CD16/32 (93) for 10 min at 4 °C. Cells were washed and stained with fluorochrome-conjugated monoclonal antibodies for 20 min at 4 °C. Subsequently, the stained cells were washed twice and resuspended in a staining buffer. Intracellular staining of Foxp3 and IL-10 was performed together using the Foxp3 stain set (eBioscience/ThermoFisher Scientific). All stained cells were evaluated using a flow cytometer (BD Bioscience, NJ, USA) and data were analyzed using FlowJo software version 10.8 (BD, Franklin Lakes, NJ, USA).

For the flow cytometry acquisition of BM-cDCs, the same electronic gate was applied to all samples and gated cells were acquired at 20,000 cells/sample. For analyses of T cells, DC, and macrophages, CD3^+^, CD11c^+^, F4/80^+^ cells, respectively, were acquired at 10,000 cells/sample.

### 4.15. Enzyme-Linked Immunosorbent Assay (ELISA) 

Cytokine levels in the culture supernatant and sera were quantified by a commercial sandwich ELISA kit. The TNF-α, IL-1β, IL-6, IL-12p70, and IL-10 ELISA kits were purchased from Biolegend and the IL-23 ELISA kit was purchased from eBioscience. The ELISA was performed following the manufacturer’s instructions. The absorbance was measured at 450 nm using a microplate reader (EPOCH2C, BioTek, Winooski, VT, USA).

### 4.16. Quantification of Anti-Double-Strand DNA Antibodies

The ELISA microplates were coated with 10 μg/mL of UltraPure^TM^ Calf Thymus DNA solution (Invitrogen, Thermofisher) at 4 °C (overnight). Next, the plates were blocked with 1% bovine serum albumin (BSA) in 1X PBS and were incubated at room temperature (1 h). Diluted samples (mouse sera) were added to the wells and the samples were incubated at room temperature (1 h) with shaking. Subsequently, the 1:4000 HRP-conjugated goat anti-mouse IgG monoclonal antibody (Biolegend) was added and the samples were incubated at room temperature (30 min). The plates were washed 4–5 times between each step. Finally, the TMB substrates were added and reactions were stopped with 2N H_2_SO_4_. The absorbance was measured at 450 nm.

### 4.17. Measurement of Serum Creatinine and Urinary Protein Creatine Index

Mouse sera and urine were diluted and creatinine levels were detected using the QuantiChrome^TM^ Creatinine Assay Kit (BioAssay Systems, Hayward, CA, USA) following the manufacturer’s instructions. The absorbance was measured at 510 nm.

For the measurement of urine protein, mouse urine was diluted and protein amounts were measured using a Bradford protein assay kit (Bio-Rad, Hercules, CA, USA). The urinary protein creatine index (PCI) was calculated using the following formula; Urinary PCI = Urinary protein (mg/L) × 10/Urinary creatinine (mmol/L).

### 4.18. Histopathology Image Analysis

The kidneys were fixed with 10% neutral buffered formalin and the fixed tissues were then embedded in paraffin. Paraffin-embedded tissues were sectioned and then stained with hematoxylin and eosin (H&E). All histological specimens were visualized under microscopy and the histological assessment was determined following a previous publication [52]. Semiquantitative evaluation of renal tubulointerstitial injury in H–E-stained slides was performed at 200X magnification in 10 randomly selected fields for each animal and the injury score was defined from the histological area with tubular epithelial swelling, loss of brush border, vacuolar degeneration, necrotic tubules, cast formation, and desquamation. The scoring of renal tubulointerstitial injury was the following: 0, damage area < 5%; 1, damage area 5–10%; 2, damage 5–10%; 3, damage 10–25%; 4, area of damage 25–50%; and 5, damage > 50%. Meanwhile, all glomeruli per slide were evaluated and glomerular injury was determined by the percentage of moderate to severe glomerular injury indicated by mesangial expansion of more than 50%, crescentic formation, and glomerulosclerosis [52].

### 4.19. Statistical Analysis

Statistical analyses were performed using IBM SPSS Statistics 29. Student’s *t*-test was used for the comparison between two groups and One-way ANOVA with Tukey’s HSD posthoc test was used for the comparison of more than two groups. For the scoring of lupus nephritis, the nonparametric Kruskal-Wallis test was used. Significant differences were considered when *p* < 0.05 and *p* < 0.001.

## 5. Conclusions

It has been well recognized that conventional immunosuppressive therapy using corticosteroids causes serious adverse effects in patients with autoimmune disease because this non-targeted therapy is involved in several cell types, including lymphoid and non-lymphoid cells. Hence, targeted therapy provides more precise treatment and fewer off-target effects. Our developed PDAMEMA-PLGA NPs possessed satisfactory sustained-release ability in company with immunomodulatory properties. PDAMEMA-PLGA NPs improved the tolerogenic effects of dexamethasone on DCs, and dexamethasone-incorporated PDAMEMA-PLGA NPs (Dex-NPs) enabled DCs to promote Treg expansion both in vitro and in vivo. Furthermore, Dex-NPs produced great therapeutic effects in *Fcgr2b*^-/-^ lupus-prone mice by mediating the selective targeting to DCs probably in a particle size-dependent manner and, as a consequence, inducing Treg expansion and antigen-specific immune tolerance. With these superior efficacies, our Dex-NPs can be further developed into an approach, in the context of DC-targeted therapy, for the reinforcement of immunosuppressive therapy and the better clinical management of SLE. 

## Figures and Tables

**Table 1:** Sizes, polydispersity indices and zeta potentials of all prepared nanoparticles.

Samples	Hydrodynamic Sizes (nm) ^a^	Polydispersity Index (pdi) ^a^	Zeta Potential (mV) ^a^
PLGA NPs	509.14 ± 11.39	0.185 ± 0.007	−24.5 ± 0.4
PDMAEMA-PLGA NPs	547.52 ± 30.04	0.214 ± 0.019	+33.2 ± 0.5
Dex-NPs	541.56 ± 51.85	0.198 ± 0.030	+32.3 ± 0.4

^a^ Hydrodynamic size, PDI, and zeta potential measured by a Zetasizer. Dex-NPs, dexamethasone-loaded PDMAEMA-PLGA NPs.

## Data Availability

No new data were created.

## Supplementary Materials

The following supporting information can be downloaded at: https://www.mdpi.com/article/10.3390/ijms24098313/s1.
