# Supplementary material for: Immunosuppressive Polymeric Nanoparticles Targeting Dendritic Cells Alleviate Lupus Disease in Fcgr2b-/- Mice by Mediating Antigen-Specific Immune Tolerance
Source: Int J Mol Sci. 2023 May 5;24(9):8313. doi: 10.3390/ijms24098313 (PMC10179670; doi:10.3390/ijms24098313)
Supplement: Supplementary file 1 [file ijms-24-08313-s001.zip › ijms-2361698-supplementary.pdf]

Supporting Information

Immunosuppressive Polymeric Nanoparticles Targeting Dendritic Cells Alleviate Lupus Disease in *Fcgr2b*$^{-/-}$ Mice by Mediating Antigen-Specific Immune Tolerance

Phuriwat Khiewkamrop [1,2,3,†], Chamraj Kaewraemruaen [4,†], Chonnavee Manipuntee [1,5], Chalathan Saengruengrit [6], Numpon Insin [1,5], Asada Leelahavanichkul [7], Warerat Kaewduangduen [1], Opor Sonpoung [8], Kasirapat Ariya-anandech [1], Nattiya Hirankarn [2,9,*] and Patcharee Ritprajak [1,10,*]

[1] Research Unit in Integrative Immuno-Microbial Biochemistry and Bioresponsive Nanomaterials, Faculty of Dentistry, Chulalongkorn University, Bangkok 10330, Thailand
[2] Center of Excellence in Immunology and Immune-Mediated Diseases, Faculty of Medicine, Chulalongkorn University, Bangkok 10330, Thailand
[3] Graduate Program in Medical Microbiology, Graduate School, Chulalongkorn University, Bangkok 10330, Thailand
[4] Department of Science and Bioinnovation, Faculty of Liberal Arts and Science, Kasetsart University, Kamphaeng Saen Campus, Nakhon Pathom 73104, Thailand
[5] Department of Chemistry, Faculty of Science, Chulalongkorn University, Bangkok 10330, Thailand
[6] Bureau of Quality and Safety of Food, Department of Medical Sciences, Ministry of Public Health, Nonthaburi 11000, Thailand
[7] Translational Research in Inflammation and Immunology Research Unit (TRIRU), Department of Microbiology, Faculty of Medicine, Chulalongkorn University, Bangkok 10330, Thailand
[8] Oral Biology Research Center, Faculty of Dentistry, Chulalongkorn University, Bangkok 10330, Thailand
[9] Immunology Unit, Department of Microbiology, Faculty of Medicine, Chulalongkorn University, Bangkok 10330, Thailand
[10] Department of Microbiology, Faculty of Dentistry, Chulalongkorn University, Bangkok 10330, Thailand
* Correspondence: nattiya.h@chula.ac.th (N.H.); patcharee.r@chula.ac.th (P.R.); Tel.: +66-2-2564132 (N.H.); +66-2-2188680 (P.R.)
† These authors contributed equally to this work.

Supplementary Materials and Methods

Cellular cytotoxicity assay

BM-cDCs ($1x10^5$ cells) were seeded in 96-well plate in 200 µl of RPMI 1640 medium (GIBCO) supplemented with 10% HI-FBS (GIBCO), 2 mM Glutamax (GIBCO), penicillin (100 IU/ml) and streptomycin (100 ug/ml) (GIBCO). The cells were incubated with PDMAEME-PLGA NPs at the concentration of 12.5, 25, 50, 100, and 200 µg/ml for 24 and 48 hours. Subsequently, one hundred microliters of the culture supernatant were removed and 20 µl of MTS solution (CellTiter 96® AQ_{ueous} One Solution Cell Proliferation Assay; Promega, WI, USA) were added into each well. Then the cells were incubated at 37°C under a humidified atmosphere containing 5% CO_2 for 3 hours and the absorbance at 490nm was measured using a microplate reader (EPOCH2C, BioTek). Unstimulated BM-cDCs were used as a negative control. The percent cell viability was calculated by the following formula; O.D. of sample x 100/average O.D. of the negative control.

Supplementary Figures

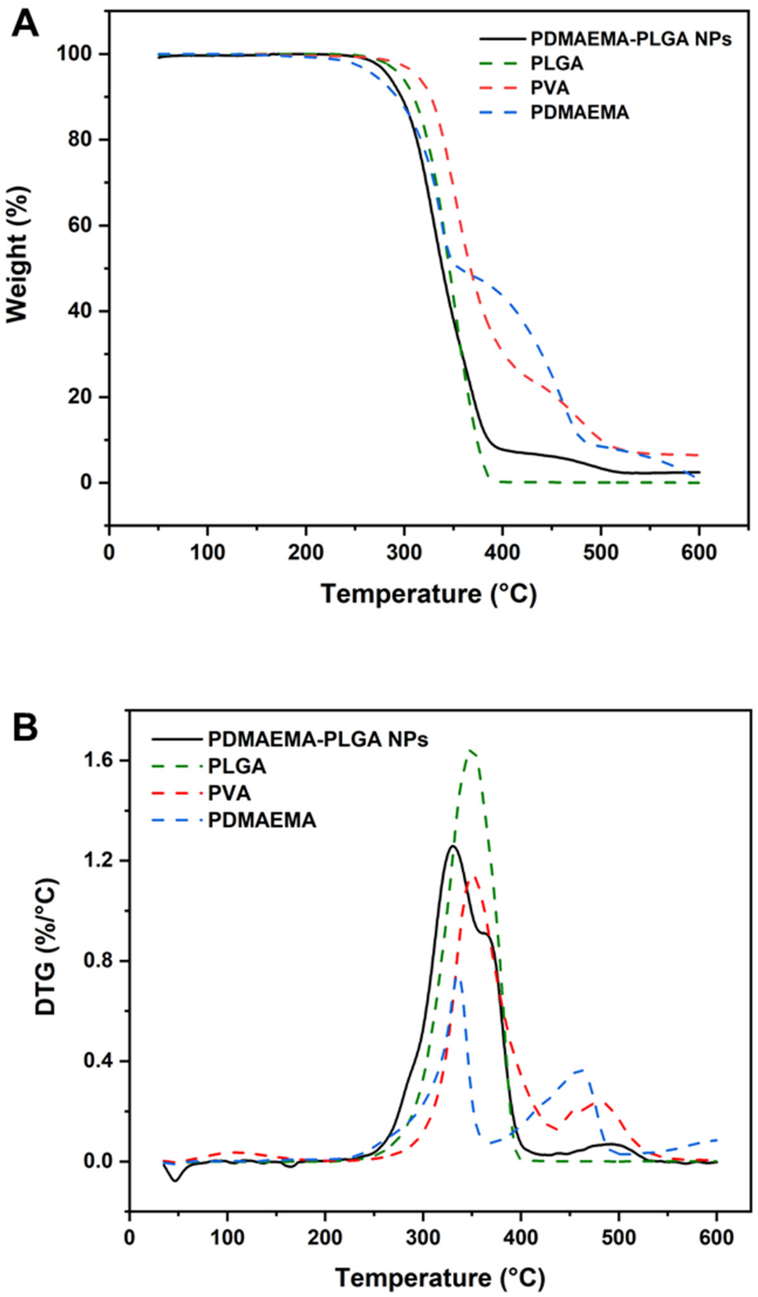

Figure S1 (A) Thermogravimetric Analysis (TGA) and (B) differential thermogravimetric analysis (DTG) of PLGA-PDMAEMA NPs compared with PLGA, PVA and PDMAEMA polymers using as starting materials. All samples were heated from 50 to 600 °C at a heating rate of 20 °C/min under a nitrogen atmosphere. PLGA, poly(lactic-co-glycolic acid). PVA, poly(vinyl alcohol). PDMAEMA, poly(2-(dimethylamino)ethyl methacrylate).

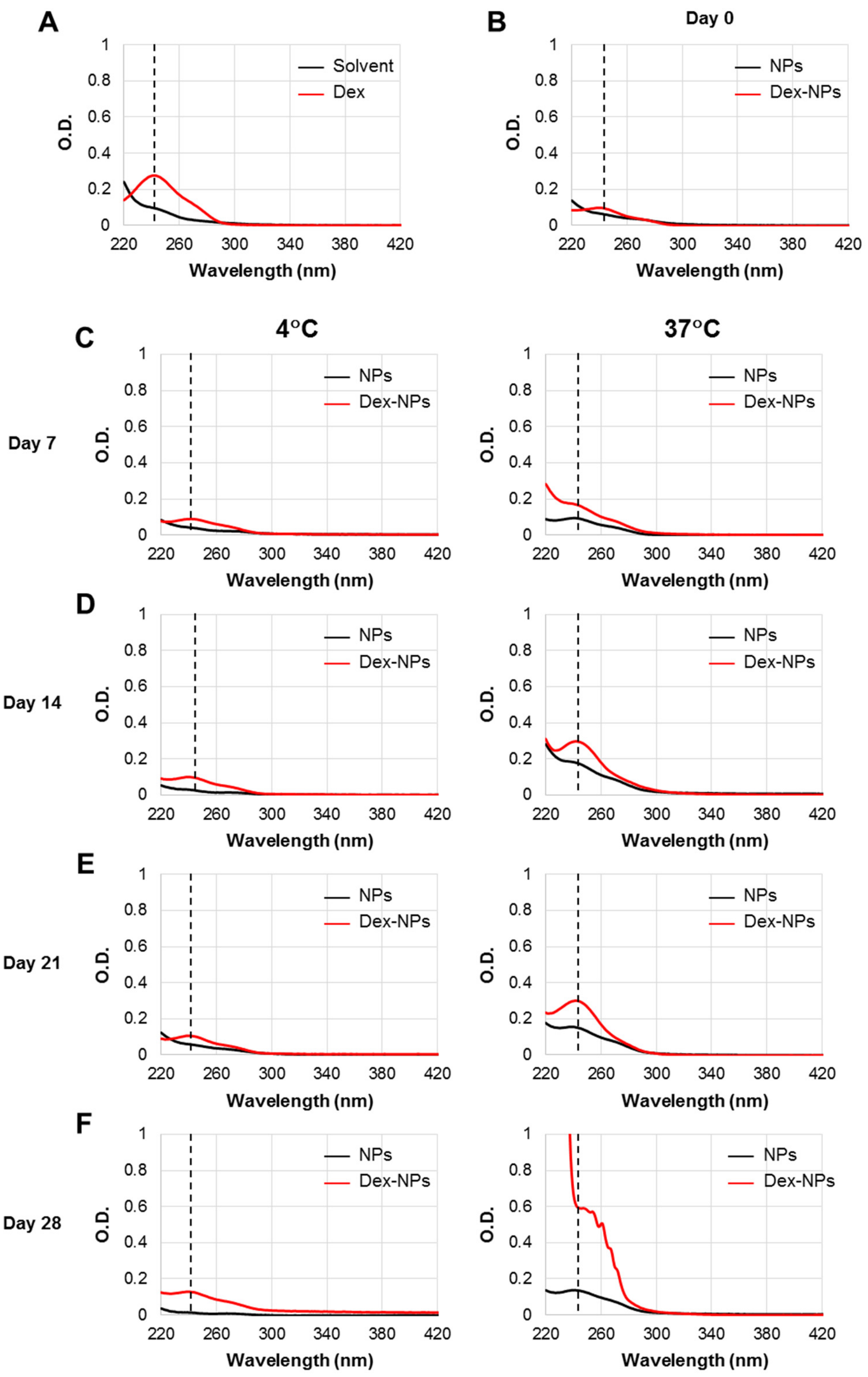

Figure S2 Spectrophotometer profiles of drug release assay. (A) Wavelength of solvent and pure dexamethasone. Measurement of free dexamethasone released from PDMAEMA-PLGA NPs at 4°C and 37°C on (B) day 0, (C) days 7, (D) days 14, (E) days 21, and (F) days 28. Dex, pure dexamethasone; NPs, PDMAEMA-PLGA NPs; Dex-NPs, dexamethasone-incorporated PDMAEMA-PLGA NPs

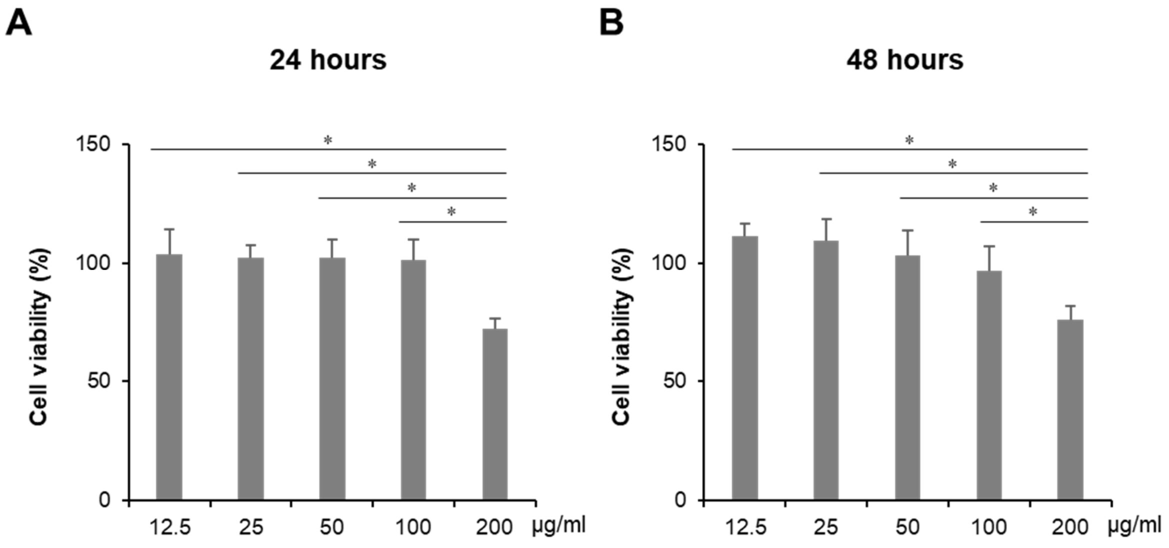

Figure S3 Cellular toxicity. BM-cDCs were treated with PDMAEMA-PLGA NPs at various concentration as indicated for (A) 24 hours, and (B) 48 hours. The cell viability was measured by MTS and the percent cell viability was calculated as described in Supplementary Materials and Methods.

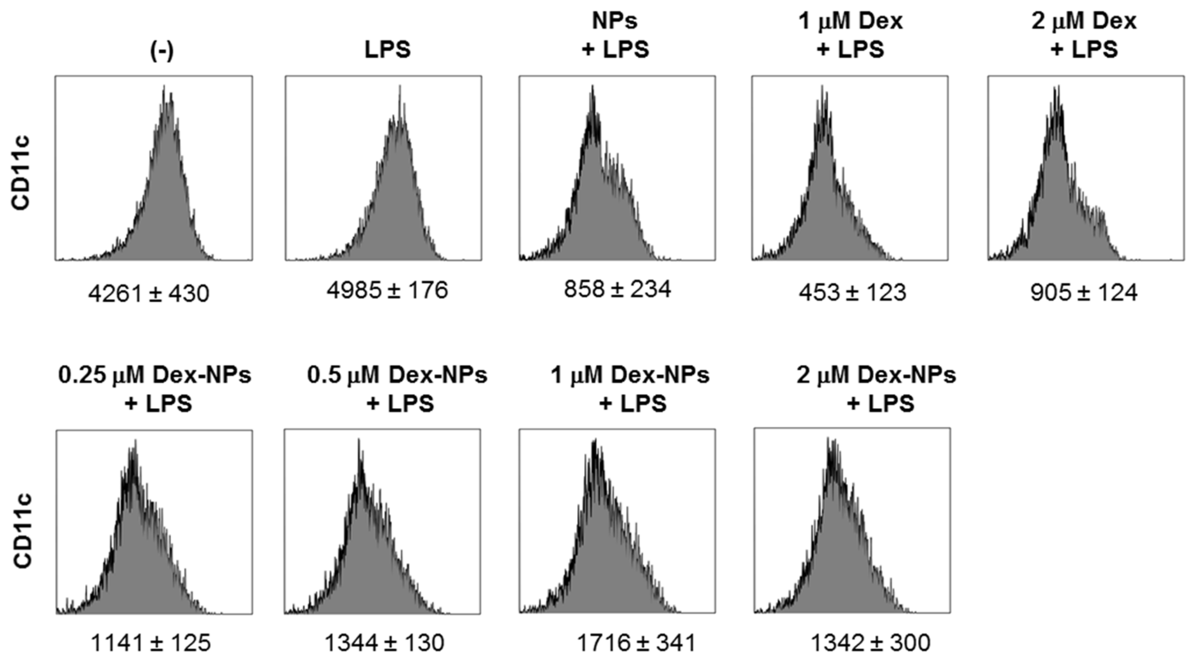

Figure S4 Histogram analysis of CD11c expression in wild-type BM-cDCs. The number indicated the average value of mean fluorescence intensity ± SD. (-), negative control (untreated and unstimulated BM-cDCs); LPS, LPS-stimulated BM-cDCs; Dex, dexamethasone; Dex-NPs, dexamethasone-incorporated PDMAEMA-PLGA NPs.

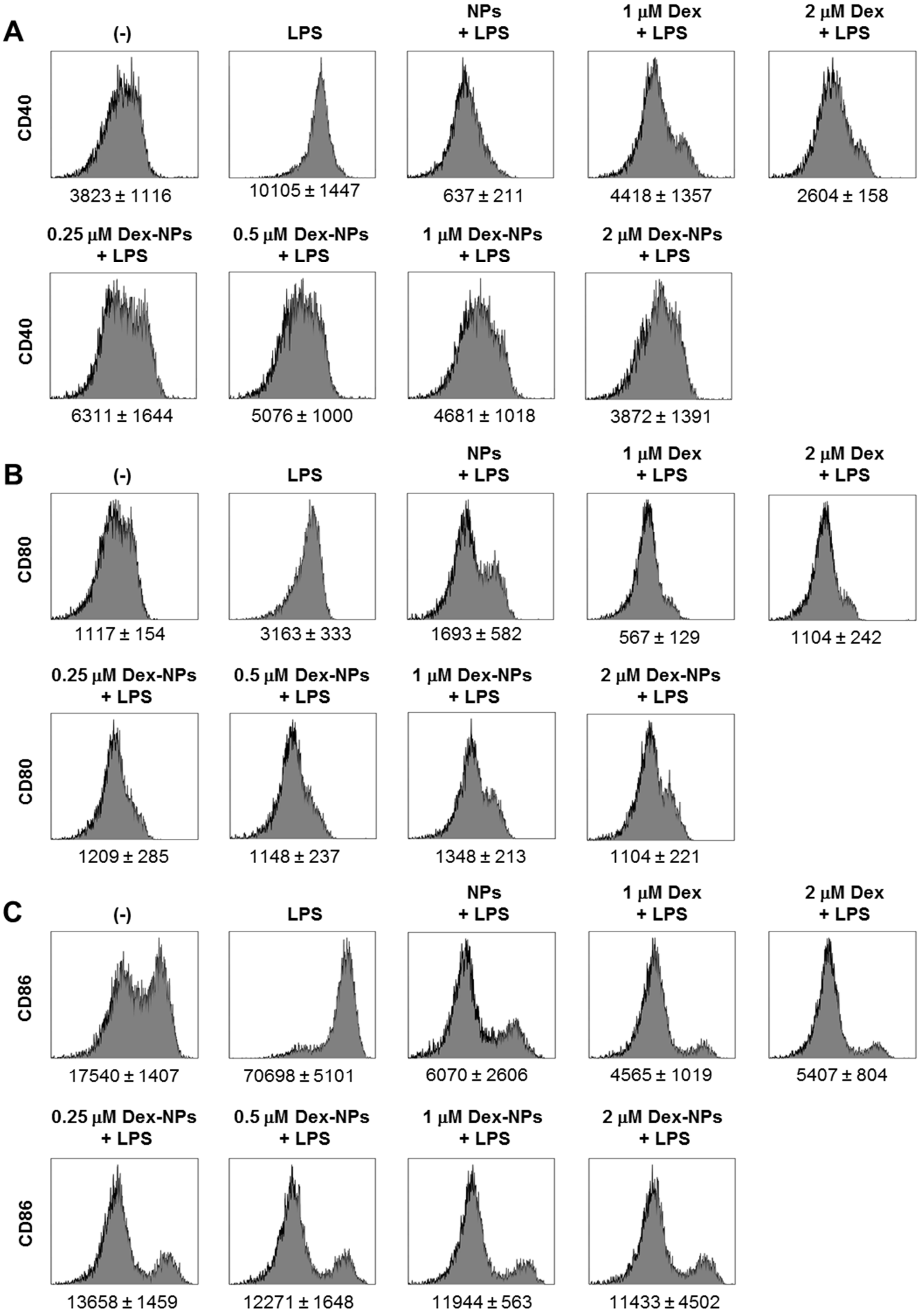

Figure S5 Histogram analysis of the expression of CD40, CD80, and CD86 in wild-type BM-cDCs. The number indicated the average value of mean fluorescence intensity ± SD. (-), negative control (untreated and unstimulated BM-cDCs); LPS, LPS-stimulated BM-cDCs; Dex, dexamethasone; Dex-NPs, dexamethasone-incorporated PDMAEMA-PLGA NPs.

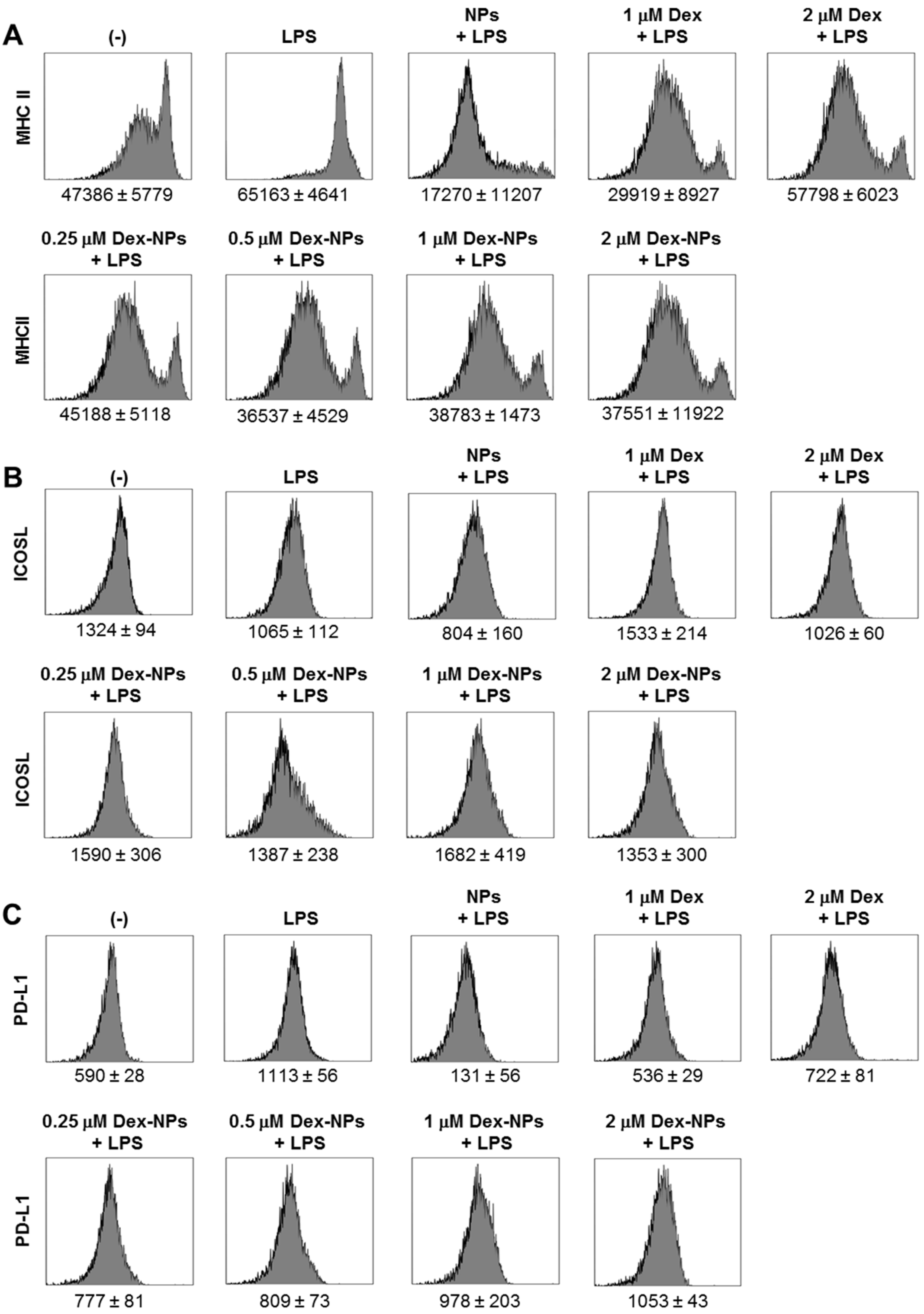

Figure S6 Histogram analysis of the expression of MHC class II, ICOSL, and PD-L1 in wild-type BM-cDCs. The number indicated the average value of mean fluorescence intensity ± SD. (-), negative control (untreated and unstimulated BM-cDCs); LPS, LPS-stimulated BM-cDCs; Dex, dexamethasone; Dex-NPs, dexamethasone-incorporated PDMAEMA-PLGA NPs.

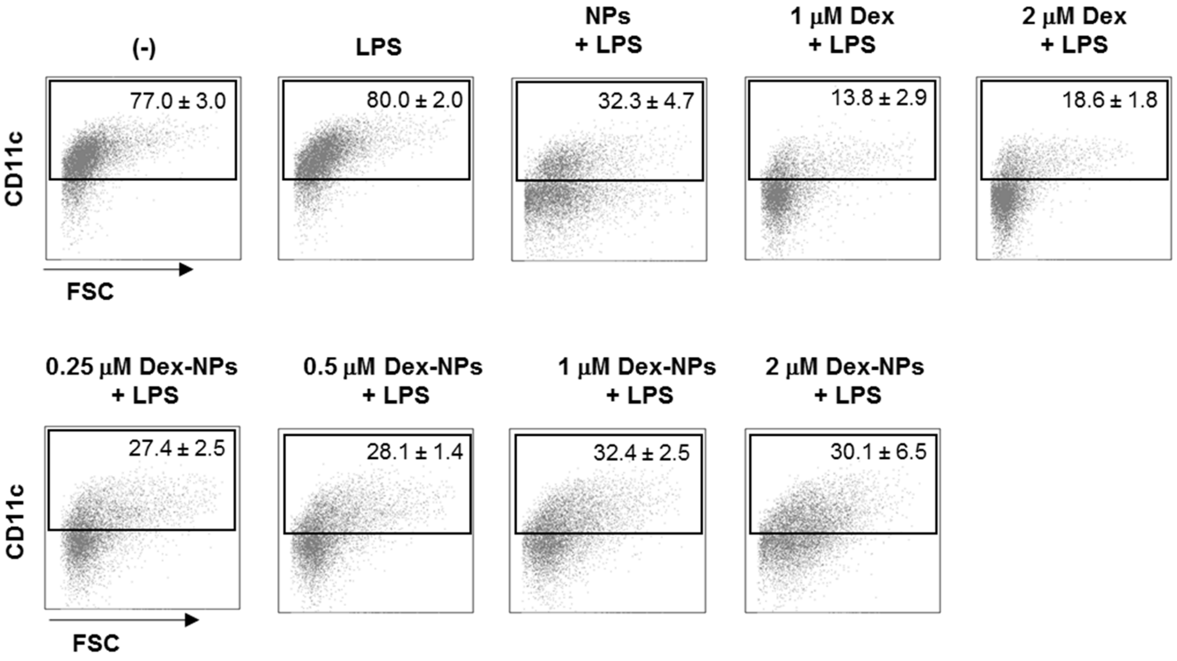

Figure S7 Dot plot analysis of CD11c⁺ population in wild-type BM-cDCs. The number indicated the average value of mean fluorescence intensity ± SD. (-), negative control (untreated and unstimulated BM-cDCs); LPS, LPS-stimulated BM-cDCs; Dex, dexamethasone; Dex-NPs, dexamethasone-incorporated PDMAEMA-PLGA NPs.

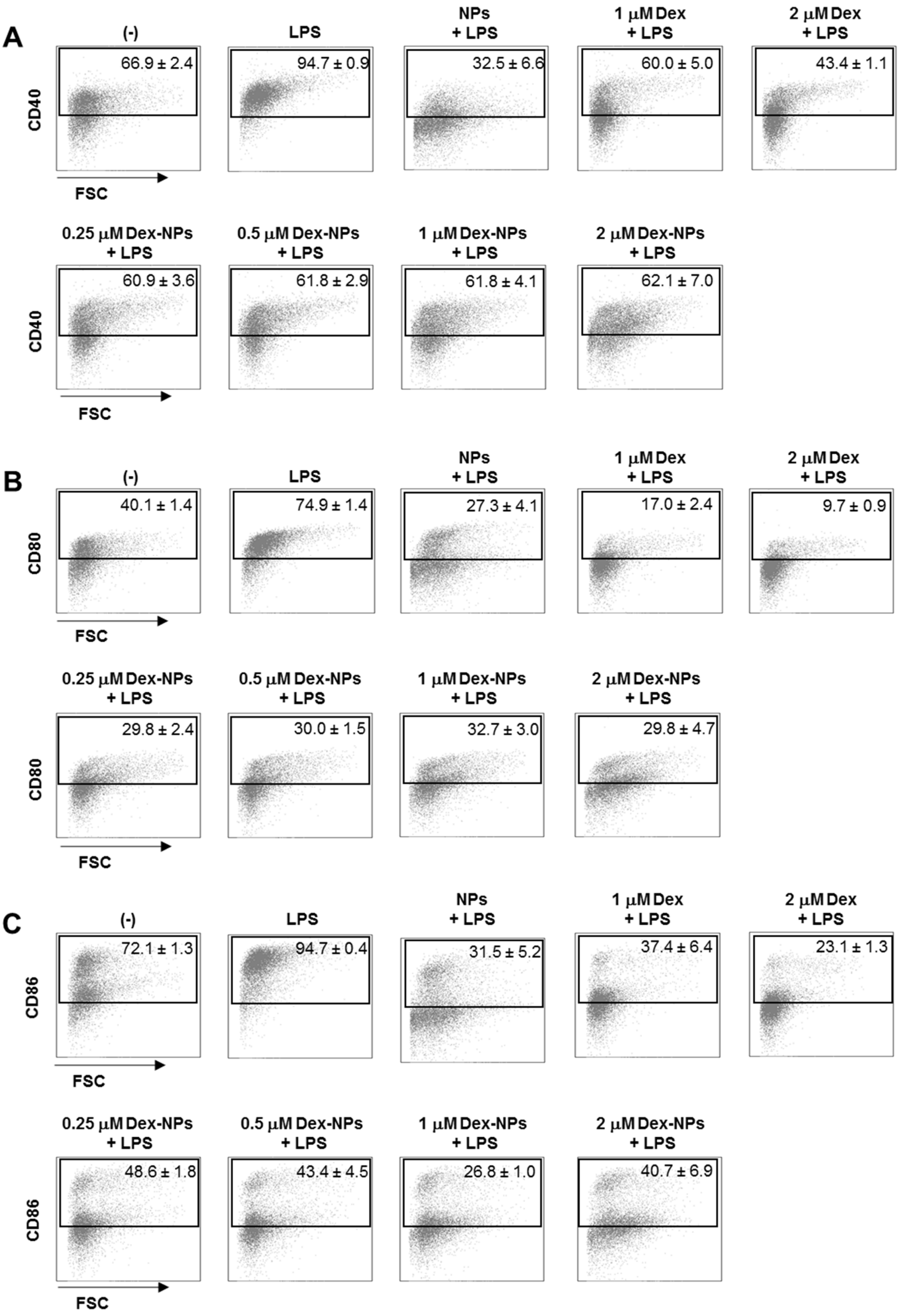

Figure S8 Dot plot analysis of CD40$^+$, CD80$^+$, and CD86$^+$ population in wild-type BM-cDCs. The number indicated the average value of mean fluorescence intensity ± SD. (-), negative control (untreated and unstimulated BM-cDCs); LPS, LPS-stimulated BM-cDCs; Dex, dexamethasone; Dex-NPs, dexamethasone-incorporated PDMAEMA-PLGA NPs.

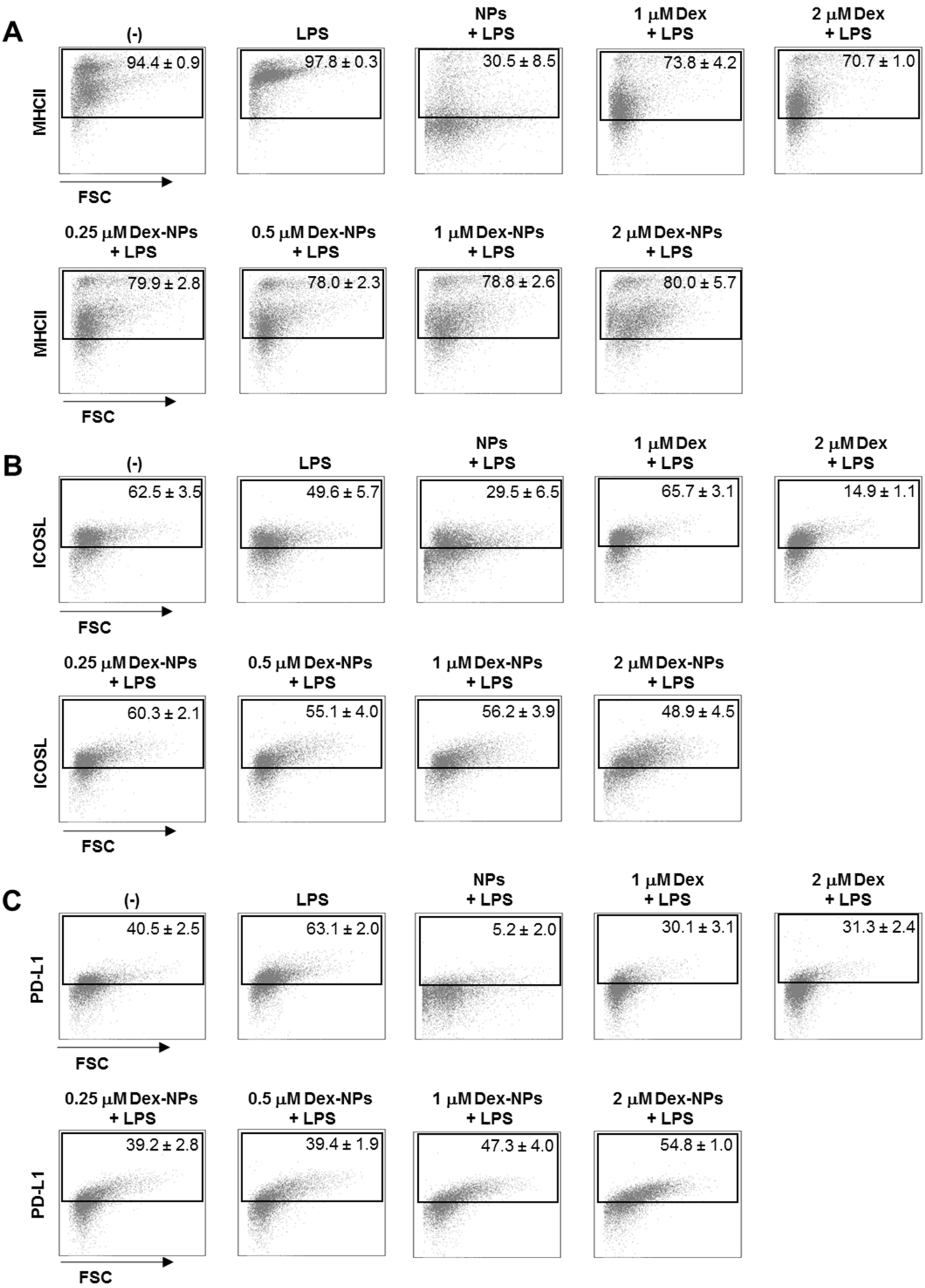

Figure S9 Dot plot analysis of MHC class II$^+$, ICOSL$^+$, and PD-L1$^+$ population in wild-type BM-cDCs. The number indicated the average value of mean fluorescence intensity ± SD. (-), negative control (untreated and unstimulated BM-cDCs); LPS, LPS-stimulated BM-cDCs; Dex, dexamethasone; Dex-NPs, dexamethasone-incorporated PDMAEMA-PLGA NPs.

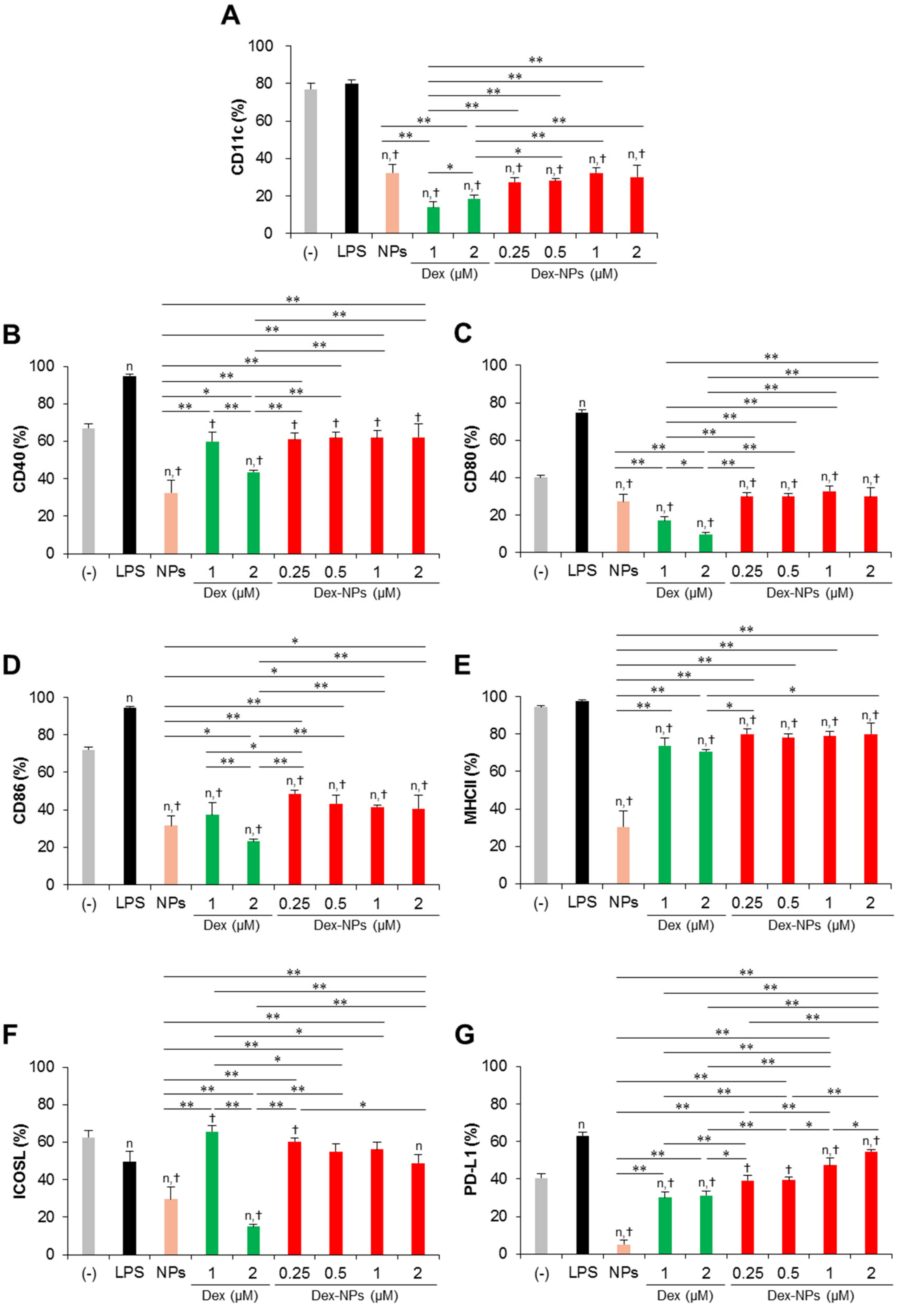

Figure S10 Analysis of activated wild-type BM-cDC population

Wild-type BM-cDCs (1x10^6 cells/well) were pre-incubated with blank PDMAEMA-PLGA NPs (55 μg, an equal amount to Dex-NPs containing 2 μM dexamethasone), dexamethasone

(1 and 2 μM), and dexamethasone-incorporated PDMAEMA-PLGA NPs containing 1 and 2 μM dexamethasone for 48 hours. Subsequently, the DCs were stimulated with 0.1 μg/ml of LPS for 24 hours. (**A**) CD11c^{+}, (**B**) CD40^{+}, (**C**) CD80^{+}, (**D**) CD86^{+}, (**E**) MHC class II^{+}, (**F**) ICOSL^{+}, and (**G**) PD-L1^{+} population were assessed by flow cytometry. $n = 5$; ^{n} $p \leq 0.05$ compared with the negative control, $^{\dagger}p \leq 0.05$ compared with LPS-stimulated BM-cDCs, $^{*}p \leq 0.05$, ** $p \leq 0.001$; (-), negative control (untreated and unstimulated BM-cDCs); LPS, LPS-stimulated BM-cDCs; Dex, dexamethasone; Dex-NPs, dexamethasone-incorporated PDMAEMA-PLGA NPs.

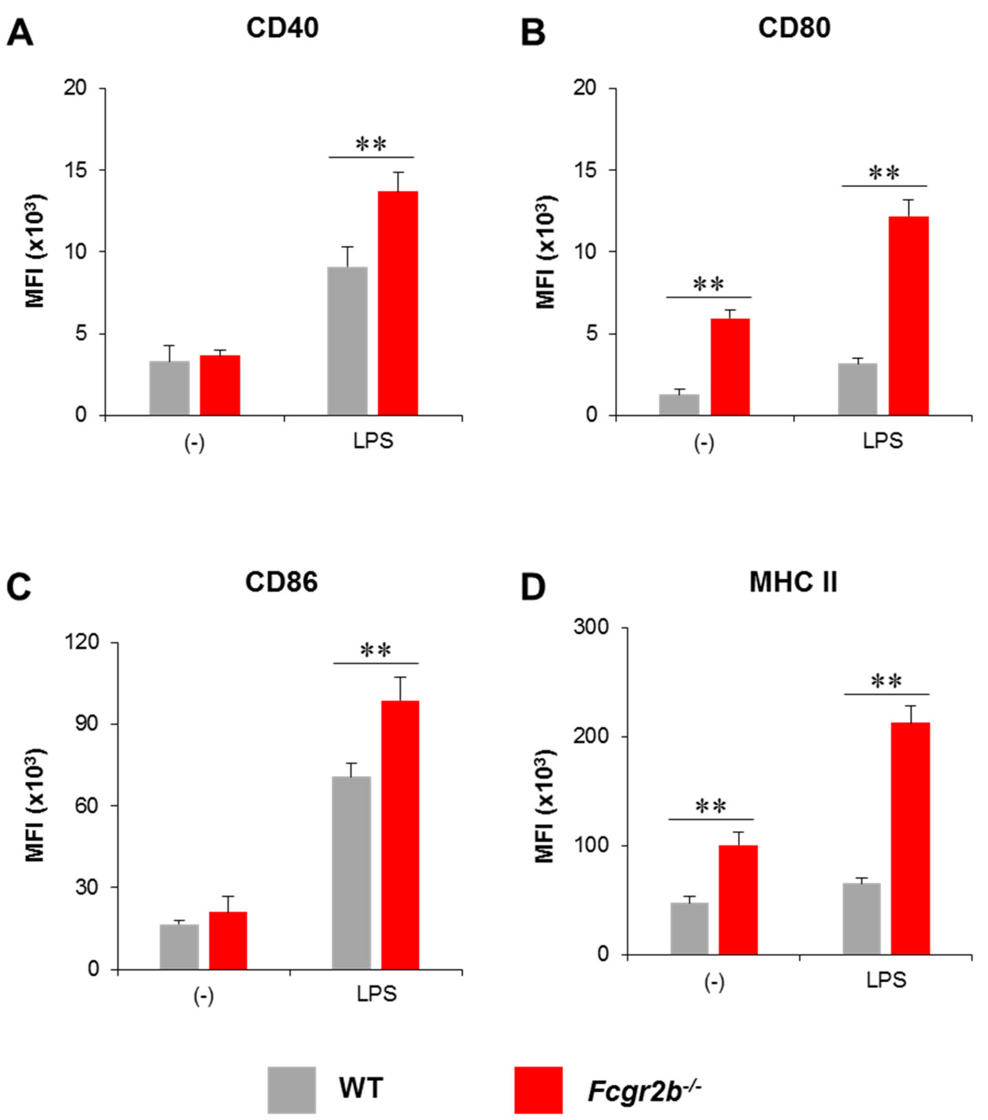

Figure S11 Comparison of DC maturation in wild-type and *Fcgr2b*$^{-/-}$ BM-cDCs. Wild-type and *Fcgr2b*$^{-/-}$ BM-cDCs ($1x10^6$ cells/well) were stimulated with 0.1 µg/ml LPS and the expression of DC maturation marker, (A) CD40, (B) CD80, (C) CD86, and (D) MHC class II was assessed by flow cytometry. $n = 5$, $*p \leq 0.05$, $**\ p \leq 0.001$; (-), negative control (unstimulated BM-cDCs); LPS, LPS-stimulated BM-cDCs; MFI, mean fluorescence intensity.

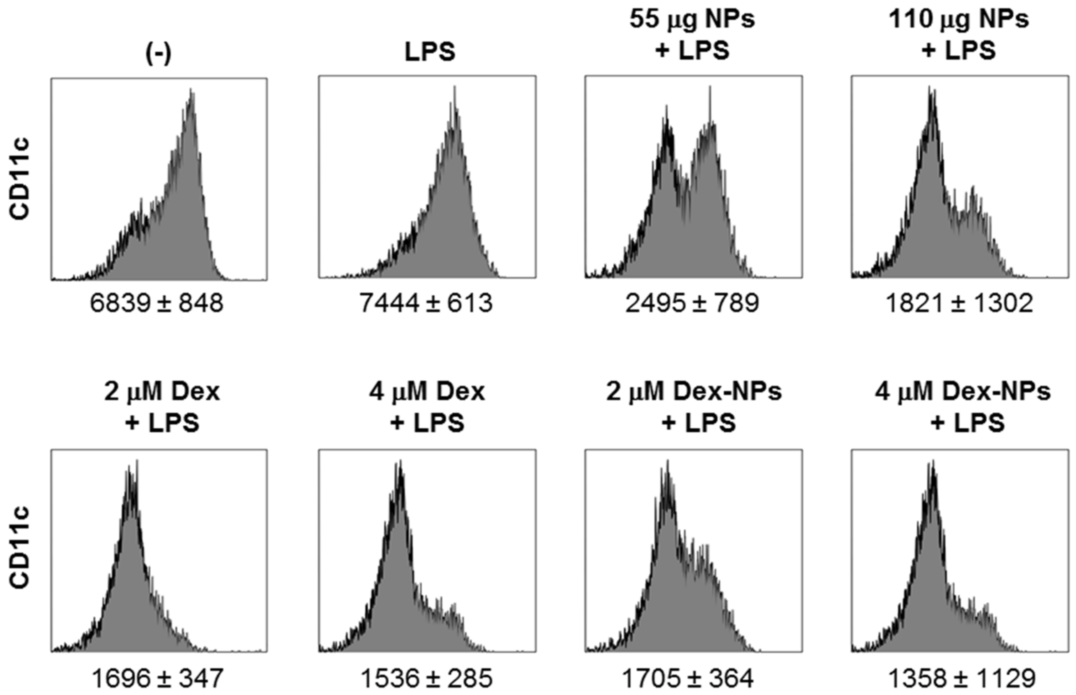

Figure S12 Histogram analysis of CD11c expression in *Fcgr2b*$^{-/-}$ BM-cDCs. The number indicated the average value of mean fluorescence intensity ± SD. (-), negative control (untreated and unstimulated BM-cDCs); LPS, LPS-stimulated BM-cDCs; Dex, dexamethasone; Dex-NPs, dexamethasone-incorporated PDMAEMA-PLGA NPs.

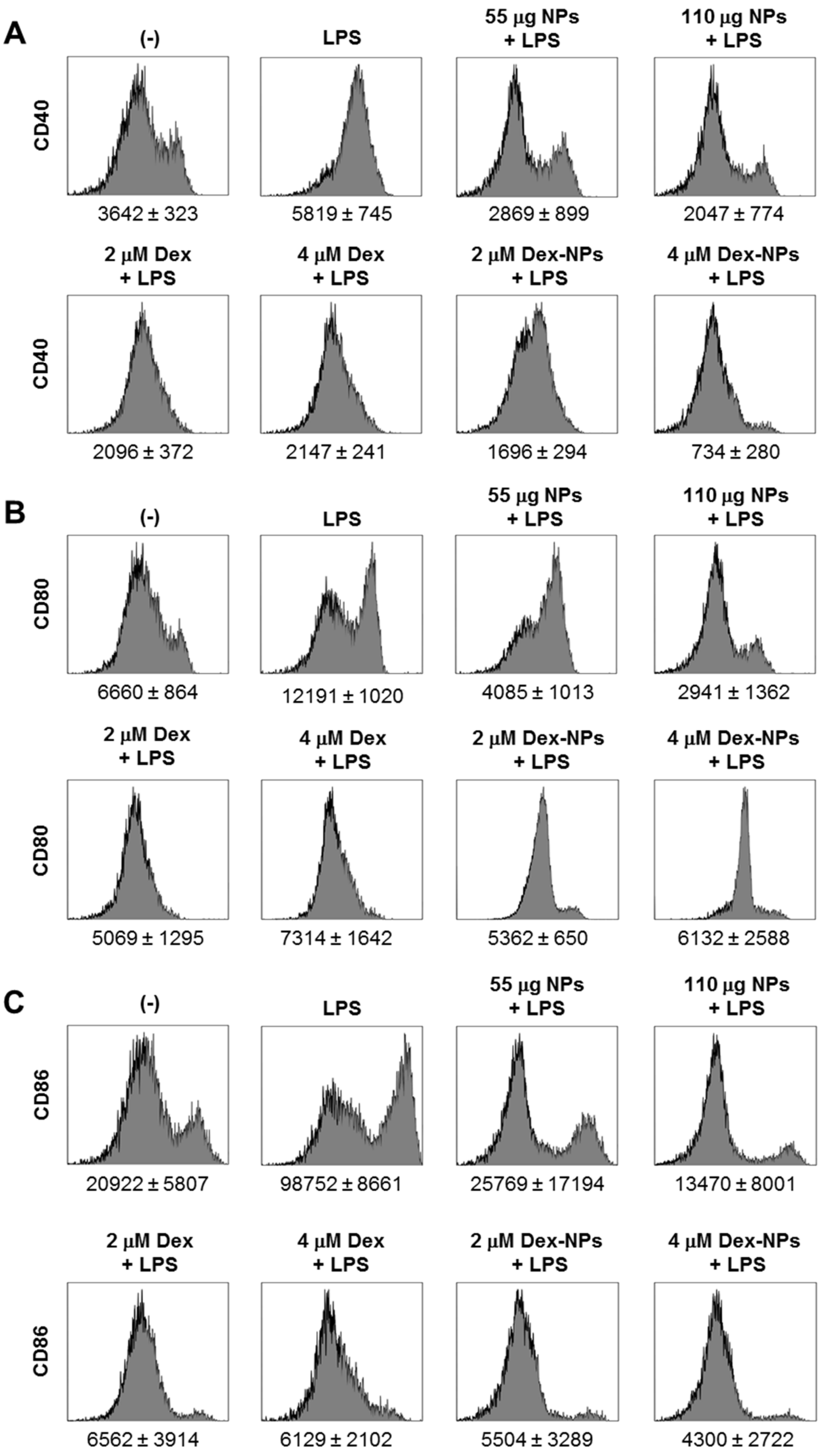

Figure S13 Histogram analysis of the expression of CD40, CD80, and CD86 in *Fcgr2b*$^{-/-}$ BM-cDCs. The number indicated the average value of mean fluorescence intensity ± SD. (-), negative control (untreated and unstimulated BM-cDCs); LPS, LPS-stimulated BM-cDCs; Dex, dexamethasone; Dex-NPs, dexamethasone-incorporated PDMAEMA-PLGA NPs.

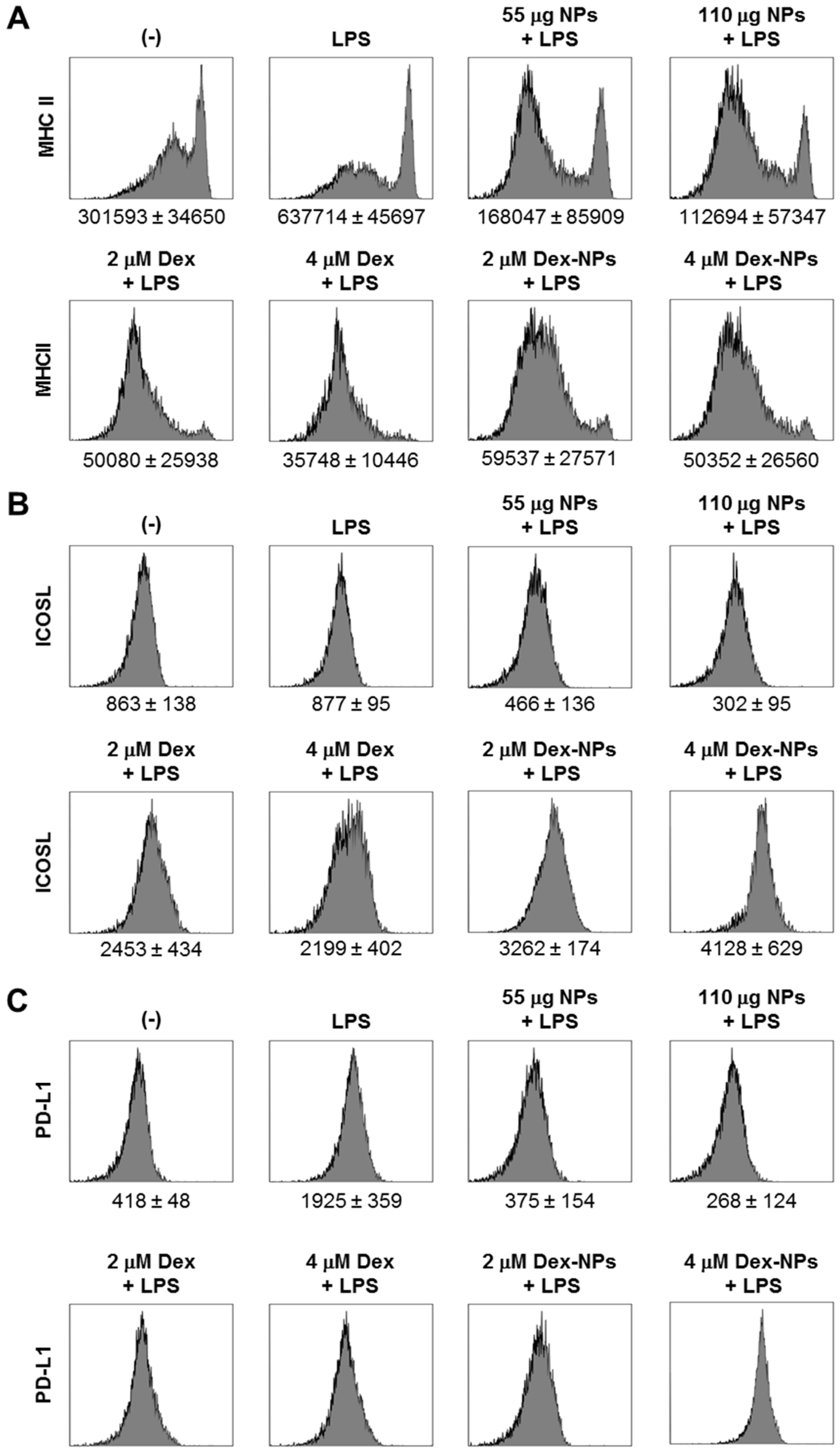

Figure S14 Histogram analysis of the expression of MHC class II, ICOSL, and PD-L1 in w *Fcgr2b*$^{-/-}$ BM-cDCs. The number indicated the average value of mean fluorescence intensity ± SD. (-), negative control (untreated and unstimulated BM-cDCs); LPS, LPS-stimulated BM-cDCs; Dex, dexamethasone; Dex-NPs, dexamethasone-incorporated PDMAEMA-PLGA NPs.

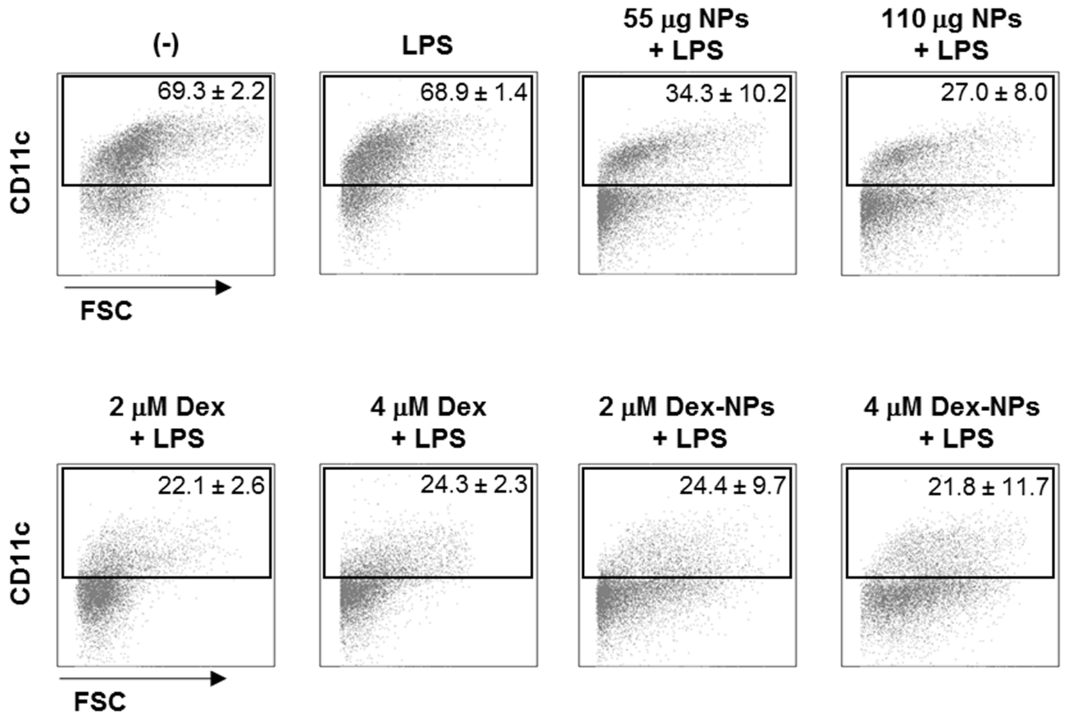

Figure S15 Dot plot analysis of CD11c$^+$ population in *Fcgr2b$^{-/-}$* BM-cDCs. The number indicated the average value of mean fluorescence intensity ± SD. (-), negative control (untreated and unstimulated BM-cDCs); LPS, LPS-stimulated BM-cDCs; Dex, dexamethasone; Dex-NPs, dexamethasone-incorporated PDMAEMA-PLGA NPs.

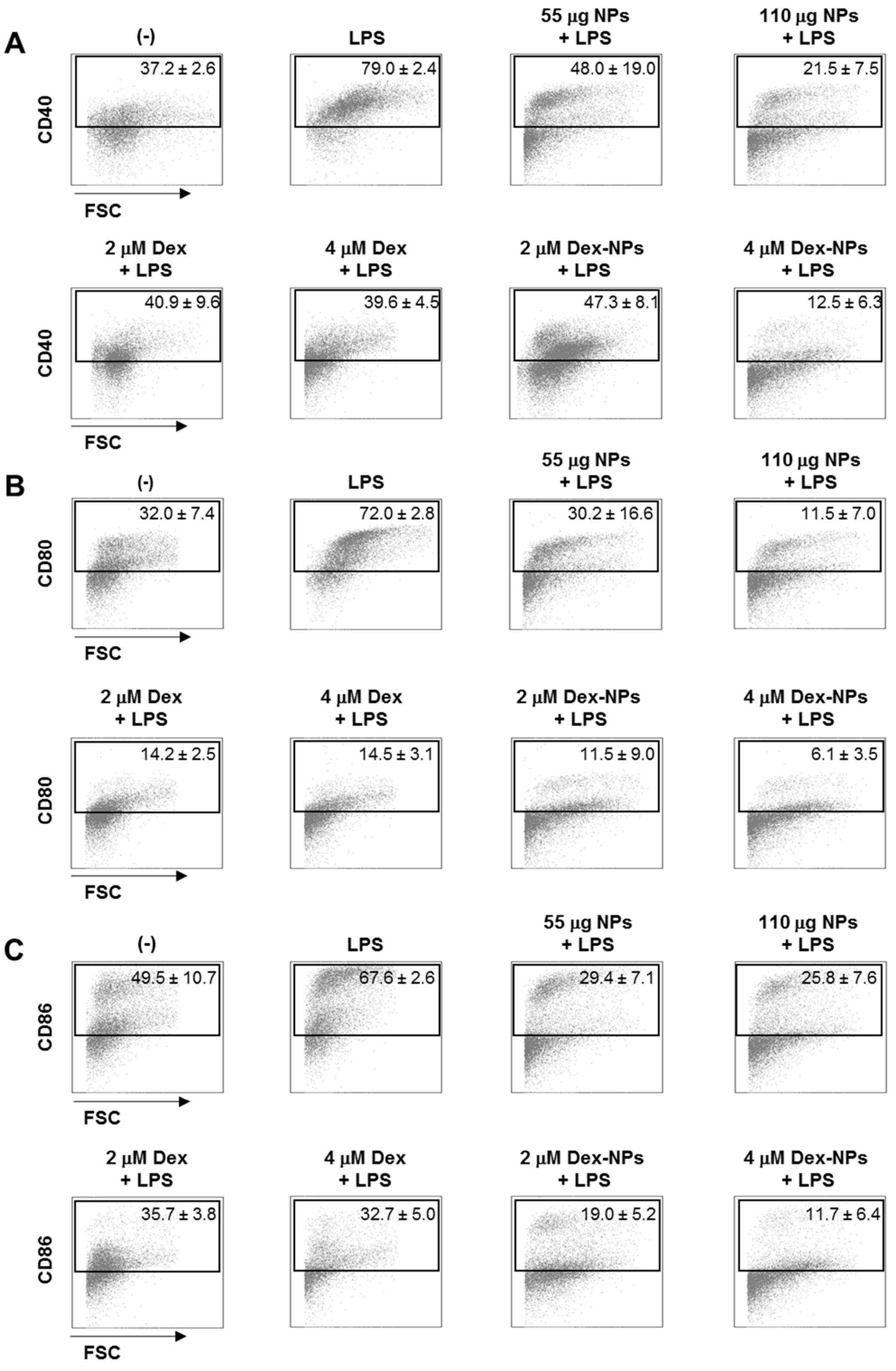

Figure S16 Dot plot analysis of CD40^{+}, CD80^{+}, and CD86^{+} population in *Fcgr2b$^{-/-}$* BM-cDCs. The number indicated the average value of mean fluorescence intensity ± SD. (-), negative control (untreated and unstimulated BM-cDCs); LPS, LPS-stimulated BM-cDCs; Dex, dexamethasone; Dex-NPs, dexamethasone-incorporated PDMAEMA-PLGA NPs.

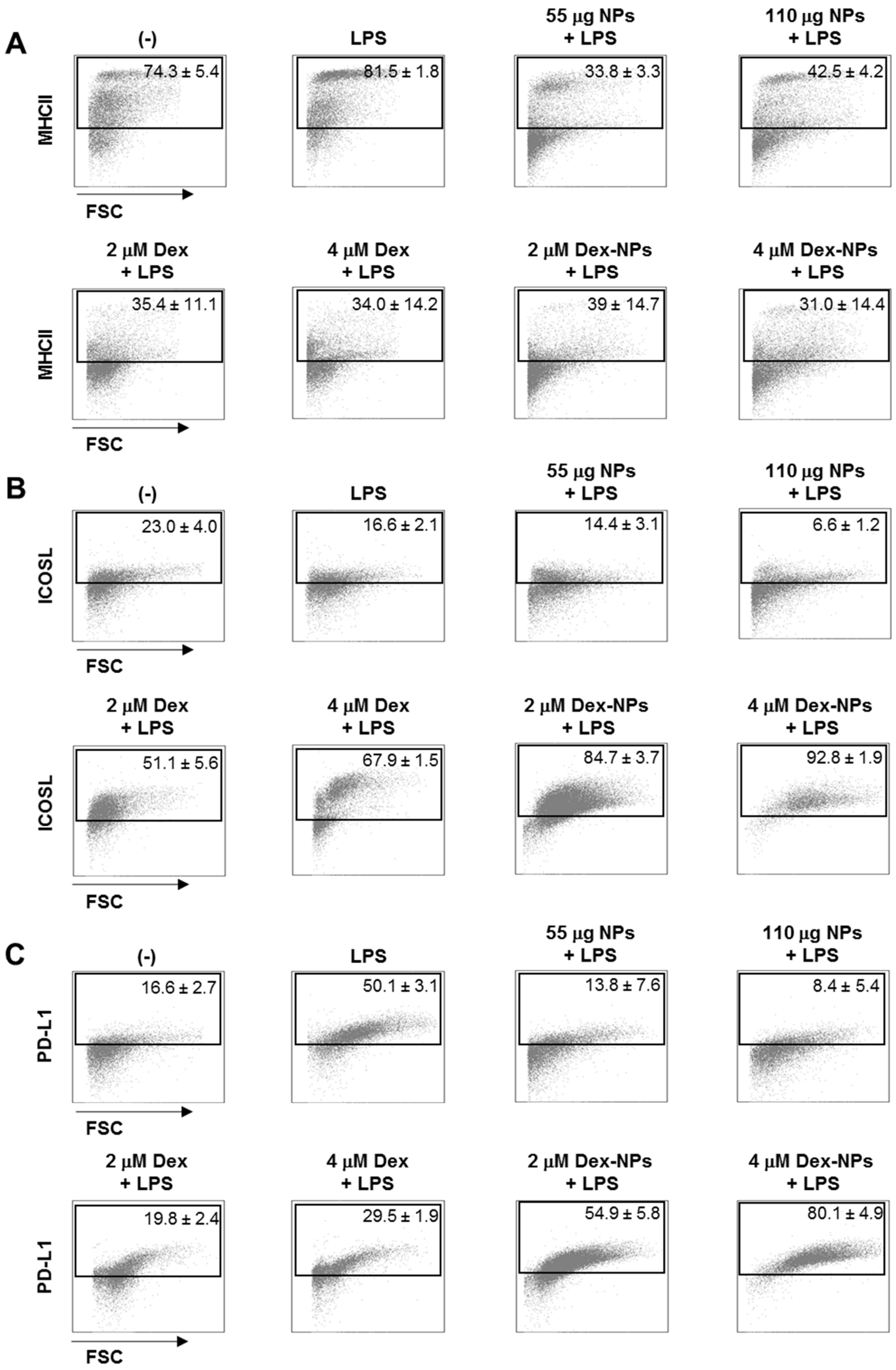

Figure S17 Dot plot analysis of MHC class II$^+$, ICOSL$^+$, and PD-L1$^+$ population in *Fcgr2b$^{-/-}$* BM-cDCs. The number indicated the average value of mean fluorescence intensity ± SD. (-), negative control (untreated and unstimulated BM-cDCs); LPS, LPS-stimulated BM-cDCs; Dex, dexamethasone; Dex-NPs, dexamethasone-incorporated PDMAEMA-PLGA NPs.

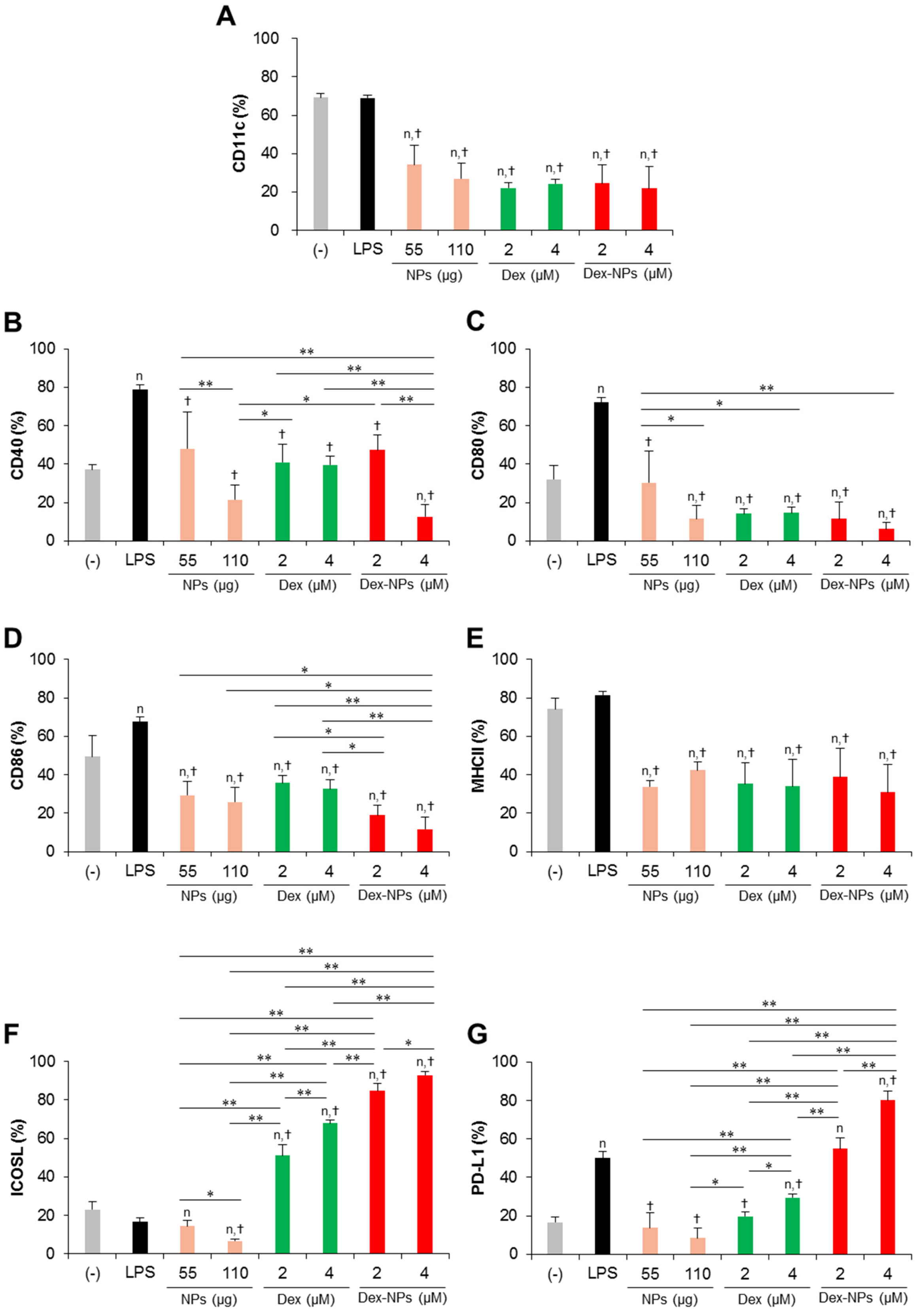

Figure S18 Analysis of activated *Fcgr2b*$^{-/-}$ BM-cDC population

Fcgr2b$^{-/-}$ BM-cDCs (1x10^6 cells/well) were pre-incubated with blank PDMAEMA-PLGA NPs (55 µg, an equal amount to Dex-NPs containing 2 µM dexamethasone), dexamethasone (1 and

2 μM), and dexamethasone-incorporated PDMAEMA-PLGA NPs containing 1 and 2 μM dexamethasone for 48 hours. Subsequently, the DCs were stimulated with 0.1 μg/ml of LPS for 24 hours. (**A**) CD11c^{+}, (**B**) CD40^{+}, (**C**) CD80^{+}, (**D**) CD86^{+}, (**E**) MHC class II^{+}, (**F**) ICOSL^{+}, and (**G**) PD-L1^{+} population were assessed by flow cytometry. $n = 5$; $^{n}p \leq 0.05$ compared with the negative control, $^{\dagger}p \leq 0.05$ compared with LPS-stimulated BM-cDCs, $^{*}p \leq 0.05$, $^{**}p \leq 0.001$; (-), negative control (untreated and unstimulated BM-cDCs); LPS, LPS-stimulated BM-cDCs; Dex, dexamethasone; Dex-NPs, dexamethasone-incorporated PDMAEMA-PLGA NPs.

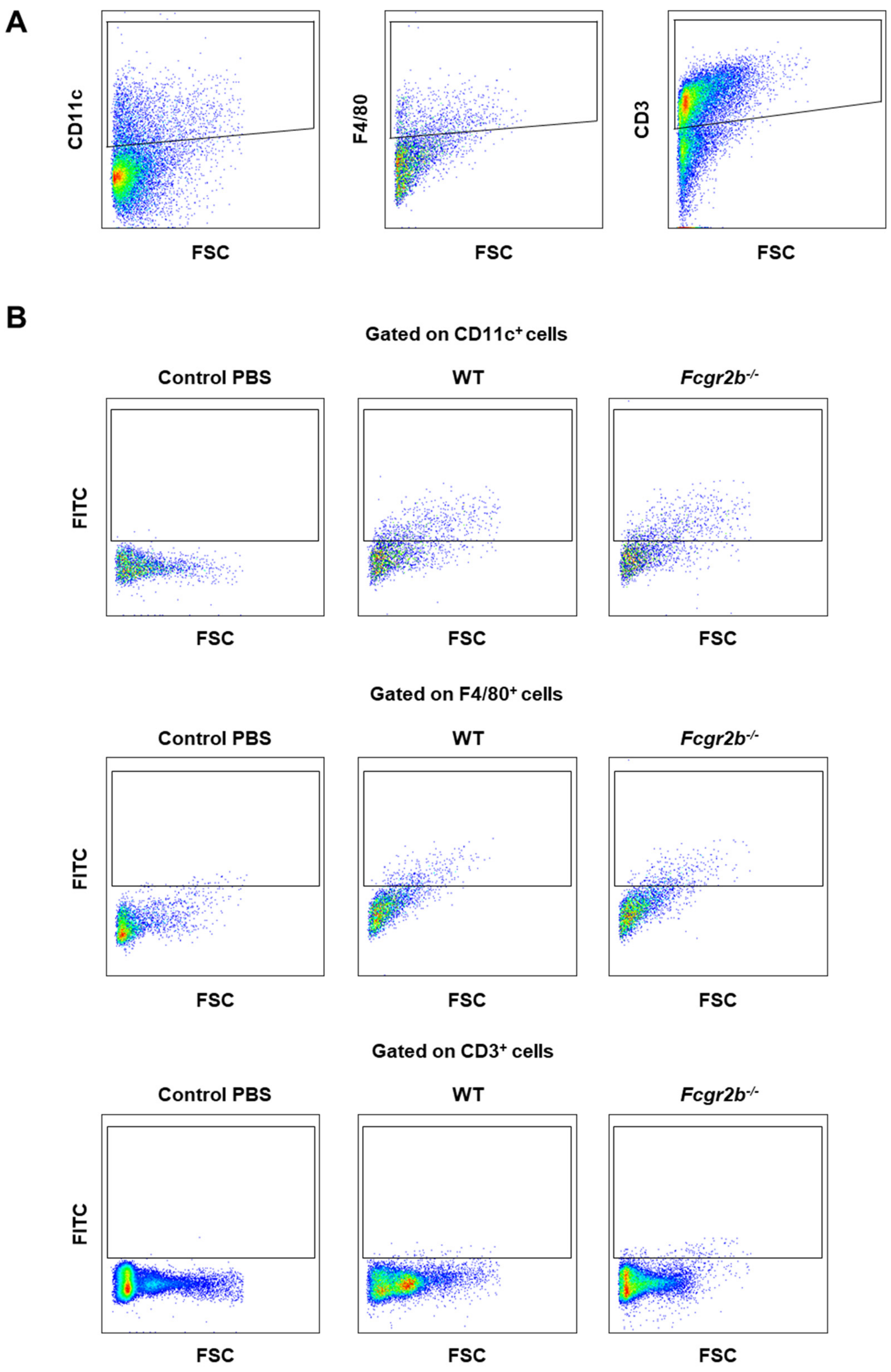

Figure S19 Flow cytometric analysis of NP uptake *in vivo*. (**A**) Analysis of CD11c$^+$ DCs, F4/80$^+$ macrophages, and CD3$^+$ T cells. (**B**) Analysis of FITC$^+$ cells in CD11c$^+$, F4/80$^+$ and CD3$^+$ cells

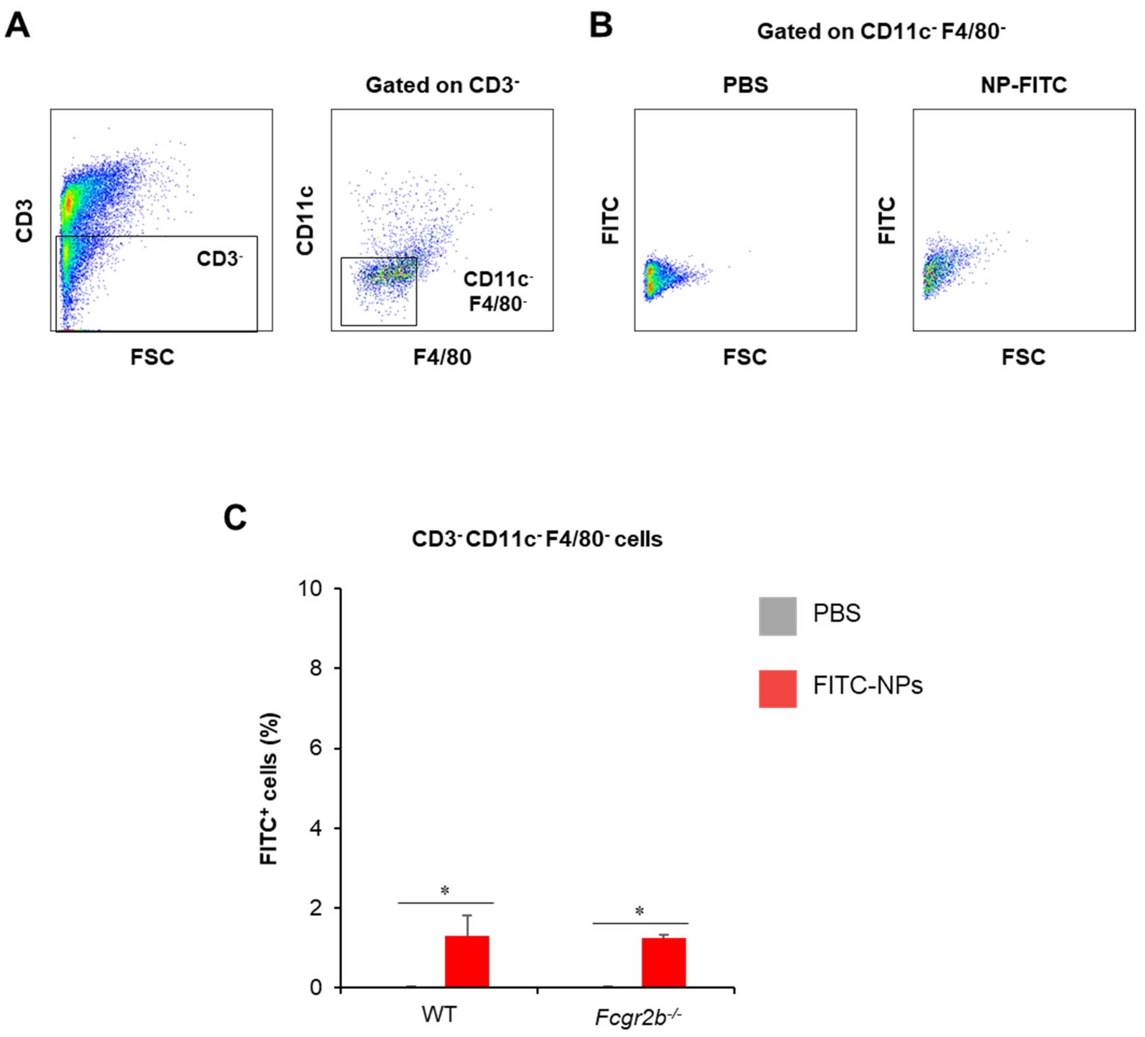

Figure S20 Flow cytometric analysis of NP uptake by non-T cells, non-Macrophage, and non-DCs. Phosphate buffer saline or FITC-tagged PDMAEMA-PLGA NPs were subcutaneously administered into wild-type and *Fcgr2b*$^{-/-}$ mice. Seventy-two hours later, (A) CD3$^-$CD11c$^-$F4/80$^-$ population in the dLNs was identified by sequential dot plot analyses and (**B**) FITC$^+$ cells in CD3$^-$CD11c$^-$F4/80$^-$ population were identified by dot plot analyses. (**C**) The proportions of FITC$^+$ cells in CD3$^-$CD11c$^-$F4/80$^-$ population. $n = 5$; $*p \leq 0.05$; PBS, the control mice that received phosphate buffer saline; FITC-NPs, mice received FITC-tagged PDMAEMA-PLGA NPs.

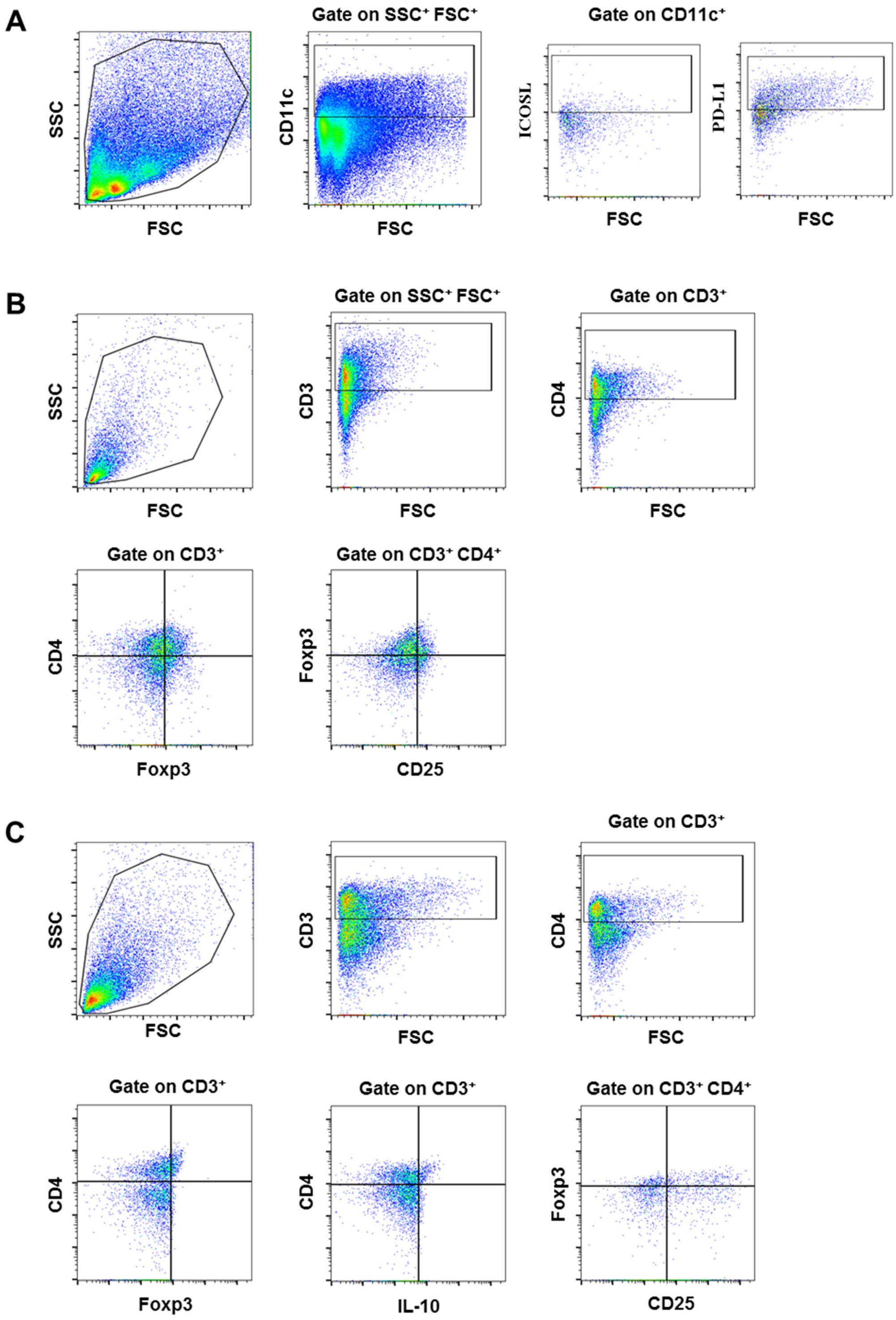

Figure S21 Flow cytometric analysis of DC and T cell population in *Fcgr2b*$^{-/-}$ lupus-prone mice. (A) Analysis of CD11+, ICOSL+, and PD-L1+ cells in dLNs (B) Analysis of CD3$^+$CD4$^+$, CD3$^+$CD4$^+$Foxp3$^+$, and CD3$^+$CD4$^+$Foxp3$^+$CD25$^+$ cells in dLNs. (C) Analysis of CD3$^+$CD4$^+$Foxp3$^+$, CD3$^+$CD4$^+$Foxp3$^+$CD25$^+$, and CD3$^+$CD4$^+$IL-10$^+$ cells in the *in vitro* restimulation assay.
